# GhMYB1 regulates SCW stage‐specific expression of the *GhGDSL* promoter in the fibres of *Gossypium hirsutum* L.

**DOI:** 10.1111/pbi.12706

**Published:** 2017-03-23

**Authors:** Vrijesh Kumar Yadav, Vikash Kumar Yadav, Poonam Pant, Surendra Pratap Singh, Rashmi Maurya, Anshulika Sable, Samir V. Sawant

**Affiliations:** ^1^ Plant Molecular Biology Laboratory CSIR‐National Botanical Research Institute Lucknow India; ^2^ Academy of Scientific and Innovative Research (AcSIR) CSIR‐National Botanical Research Institute Lucknow India

**Keywords:** *Gossypium hirsutum*, GhMYB1, GDSL promoter, cotton fibre development, site‐directed mutagenesis, secondary cell wall biosynthesis

## Abstract

Secondary cell wall (SCW) biosynthesis is an important stage of the cotton fibre development, and its transcriptional regulation is poorly understood. We selected the *Gossypium hirsutum GDSL* (*GhGDSL*) *lipase/hydrolase* gene (CotAD_74480), which is expressed during SCW biosynthesis (19 through to 25 days postanthesis; DPA), for study. T_1_‐transgenic cotton lines expressing the β‐glucuronidase (*gus*) reporter under the control of a 1026‐bp promoter fragment of *GhGDSL* (P_G__h_ _GDSL_) showed 19 DPA stage‐specific increase in GUS expression. 5′ deletion indicated that the 194‐bp fragment between –788 and –594 relative to the transcription start site was essential for this stage‐specific expression. Site‐directed mutagenesis of eight transcription factor binding sites within P_G__h_ _GDSL_ demonstrated that the MYB1AT motif (AAACCA) at –603/–598 was critical for the 19 DPA‐specific reporter gene expressions. Yeast one‐hybrid (Y1H) analysis identified nine proteins, including GhMYB1 (CotAD_64719) that bound to the P_G__h_ _GDSL_ promoter. Further, Y1H experiments using the 5′ promoter deletions and individually mutated promoter motifs indicated that GhMYB1 interacted with P_G__h_ _GDSL_ at MYB1AT sequence. *GhMYB1* was expressed specifically in fibre from 19 DPA, overlapping with the sharp rise in *GhGDSL* expression, indicating that it could regulate *GhGDSL* during fibre development. Analysis of genes co‐expressed with *GhMYB1* showed that it potentially regulates a number of other 19–25 DPA‐specific genes in networks including those functioning in the cell wall and precursor synthesis, but not the major polysaccharide and protein components of the fibre SCW. *GhGDSL* and its promoter are therefore potential tools for the improvement of cotton fibre quality traits.

## Introduction

Cotton fibre is a single‐celled structure initiated on the epidermal surface of an ovule and matures over four developmental stages, namely initiation, elongation, SCW synthesis and maturation (Basra and Malik, 1984). A single mature cotton fibre consists of the thin outer primary cell wall (PCW), an inner thick SCW and a central lumen. The most desirable agronomical traits of cotton fibres are its length, strength and fineness. The SCW controls the strength and fineness of fibres through the extent of synthesis and deposition of cellulose, the main polysaccharide component of the SCW. Among the different developmental stages of the fibre, initiation is the best understood at the transcriptional level. Although several transcription factor types are reported to play a role in cotton fibre development, the MYB transcription factors are among the most prolific. *GhMYB25*,* GaMYB2* and *GhMYB109* have been shown to control steps in fibre initiation and elongation (Deng *et al*., 2011; Pu *et al*., 2008; Walford *et al*., 2011; Wang *et al*., 2004). Cotton fibre SCW biosynthesis is characterized by the expression of genes such as *GhGlcAT1* (Wu *et al*., 2007) and *GhRLK1* (Li *et al*., 2005b), but predominantly the stage‐specific cellulose synthases such as *GhCesA4*,* GhCesA7* and *GhCesA8* (Tuttle *et al*., 2015). Recently, it has been reported that *GhKNL1* (KNOTTED1‐LIKE) is also expressed during SCW deposition (Gong *et al*., 2014). Although there are several genes that are expressed specifically during SCW synthesis, the transcription regulation of TFs involved in cotton fibre SCW biosynthesis still lacks detail investigation. However, MYB1 (R2R3‐MYB) has been shown to regulate SCW biosynthesis in *Pinus taeda* (Bomal *et al*., 2008) and cell wall integrity in fungi (Dong *et al*., 2015) so may be a potential candidate for a role in fibre SCW synthesis.

*GDSL lipases* are a very large gene family belonging to SGNH superfamily in plants (Akoh *et al*., 2004) and are conserved from microbes through to plants. More than a thousand members of *GDSL lipase* have been reported, but their functional roles in plant development and physiology are poorly explored. *Trichome birefringence* (*TBR*) in *Arabidopsis* possesses a GDSL esterase/lipase domain and plays a role in the cellulose deposition and the synthesis of SCW through the esterification of pectin compounds (Bischoff *et al*., 2010). In *Pseudomonas aeruginosa*, these lipases hydrolyse the esters of long‐chain fatty acids and acylglycerols (Wilhelm *et al*., 1999). In plants, *GDSL lipases* are involved in many developmental processes (Takahashi *et al*., 2009), seed germination morphogenesis (Clauss *et al*., 2008; Katavic *et al*., 2006; Kondou *et al*., 2008), defence (Kwon *et al*., 2009; Oh *et al*., 2005), abiotic and biotic stress (Hong *et al*., 2008; Kim *et al*., 2008) and responses to the hormones ethylene and auxin (Lee *et al*., 2009). *GDSL lipases* are expressed in cotton fibres (Nigam *et al*., 2013); however, their exact role in fibre development is not yet known.

Greater understanding of the genes, upstream regulatory elements and transcription factors that regulate fibre‐specific gene expression will be indispensable for enhancing fibre traits in the longer term. Genetic engineering for better fibres (longer, stronger and finer) will require stage‐specific promoters for manipulating those fibre properties. Several promoters have already been reported that regulate gene expression in cotton fibres during the initiation and elongation stages (Delaney *et al*., 2007; Hussey *et al*., 2011; Larkin *et al*., 1996; Li *et al*., 2002, 2009; Ma *et al*., 1995; Ni *et al*., 2008; Song and Allen, 1997; Wang *et al*., 2004; Wu *et al*., 2009). The promoter of *GaRDL1*,* GhTUB1* and *GhMYB25*, for example, is active early during the initiation stage (Li *et al*., 2002; Machado *et al*., 2009; Wang *et al*., 2004), while the glucuronosyltransferase promoter (P_GhGlcAT1_) has been shown to be highly active during fibre elongation (Wu *et al*., 2007). The fibre‐specific lipid transfer protein (*FSltp4*) promoter has also been reported to be active during fibre elongation, and its upstream gene has been shown to be functional during the synthesis of fibre cutin (Delaney *et al*., 2007). *GhACTIN1* is another gene expressed in fibre, and its promoter has been shown to be elongation‐specific (Li *et al*., 2002). Promoters for the genes, *GhLTP3*,* GhDET2*,* GaMYB2* and *GhMYB109*, have also been reported to be active during initiation and some of them continue to be active until the elongation stage (Liu *et al*., 2000; Luo *et al*., 2007; Pu *et al*., 2008; Wang *et al*., 2004). There are other promoters, such as P_GhRING1_, that are active in all the stages of cotton fibre development, starting from initiation through to the SCW stage (Ho *et al*., 2010). However, there are no reports of a truly SCW stage‐specific promoter and its detailed molecular characterization.

The present work describes the cloning and characterization of promoter regulatory sequences of SCW biosynthesis stage‐specific *GDSL lipase/hydrolase* from cotton. Our analysis demonstrates that P_GhGDSL_ is regulated by the GhMYB1 transcription factor that interacts with P_GhGDSL_ at a MYB1AT motif. We also propose that MYB1 is involved in the regulation of a broader gene network that is expressed during the SCW biosynthesis from around 19 DPA, so these genes and promoters provide potential new targets for engineering to improve fibre quality attributes.

## Results

### *GhGDSL* has SCW stage‐specific expression during cotton fibre development

**Figure 1:**
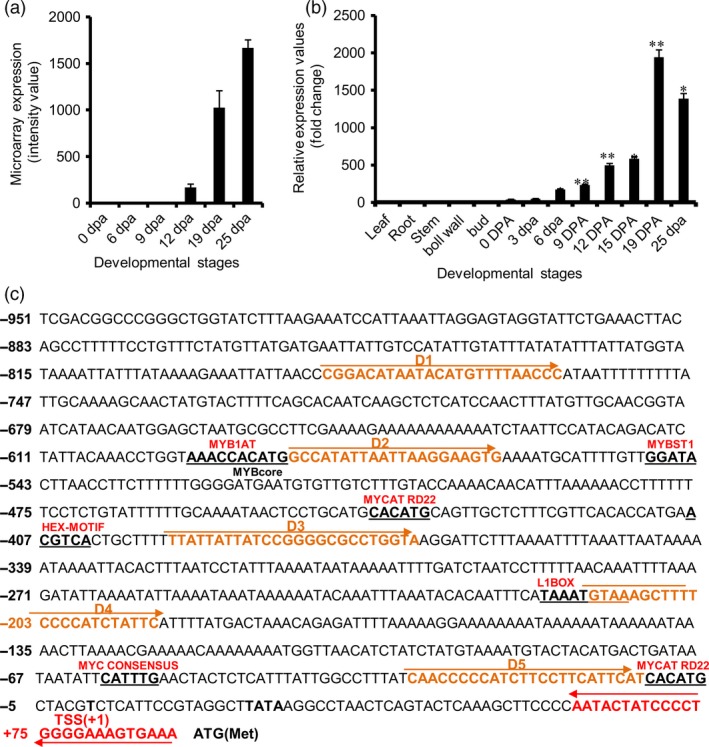
Sequence of the *GhGDSL* promoter and validation of its stage‐specific expression. (a) Microarray expression profile (average of five genotypes: 703, 737, 783, 777 and 725) of *GhGDSL* gene (*Ghi*.8746.2.A1_x_at) at the different cotton fibre development stages (0, 6, 9, 12, 19 and 25 DPA) using cotton Affymetrix chip data. (b) Quantitative RT‐PCR analysis of *GhGDSL* gene expression in the cotton fibre development stages (0–25 DPA): the normalization of the gene expression was carried out using the cotton ubiquitin gene. The various stages of fibre development are indicated in DPA (day post anthesis). The asterisk indicates statistical analysis by *t*‐test (*P‐values <0.05 and **P‐values <0.01). The error bars represent ±SE (standard error) of three independent repeats. (c) Sequence analysis of P_G__h_ _GDSL_: The putative *cis*‐regulatory elements (REs) are underlined. Transcription start site (TSS) is marked as +1 and –603/–598, –600/–594, –558/–553, –450/–445, –418/–413, –226/–219, –67/–62, –11/–6 represent the position of *cis*‐REs on the promoter.

Our previous microarray data (Nigam *et al*., 2013) based on five genotypes and six cotton development stages suggested that a *GhGDSL* (Ghi.8746.2.A1_x_at) was preferentially expressed during SCW deposition stage (19 DPA and 25 DPA) of fibre development (Figure 1a). We decided to use *GhGDSL* promoter to delineate the potential gene regulatory networks that might controls SCW formation. The expression of *GhGDSL* was further verified by qRT‐PCR using cDNA samples prepared from 0, 3, 6, 9, 12, 15, 19 and 25 DPA stages fibres and from leaf, stem, root, buds and cotton boll coat to confirm its specificity. The qRT‐PCR showed fibre‐specific expression of *GhGDSL* with negligible expression in all the other tissues tested. Expression of *GhGDSL* was significantly higher at the 19 and 25 DPA stages (Figure 1b), similar to that observed in the microarrays. These results confirmed the fibre and SCW stage‐specific expression of *GhGDSL*. We also verified that the 19 and 25 DPA stages actually represent stages of SCW deposition by measuring the total cellulose content in each of the different fibre samples and saw significant cellulose deposition after 15 DPA (Figure S6). The phylogenetic analysis of *GhGDSL* (gene id CotAD_74480) with the known *GDSL* genes in the *Arabidopsis* genome demonstrated significant homology with the *Arabidopsis* APG2.ARATH GDSL lipase/hydrolase protein (Figure S7).

**Figure 2:**
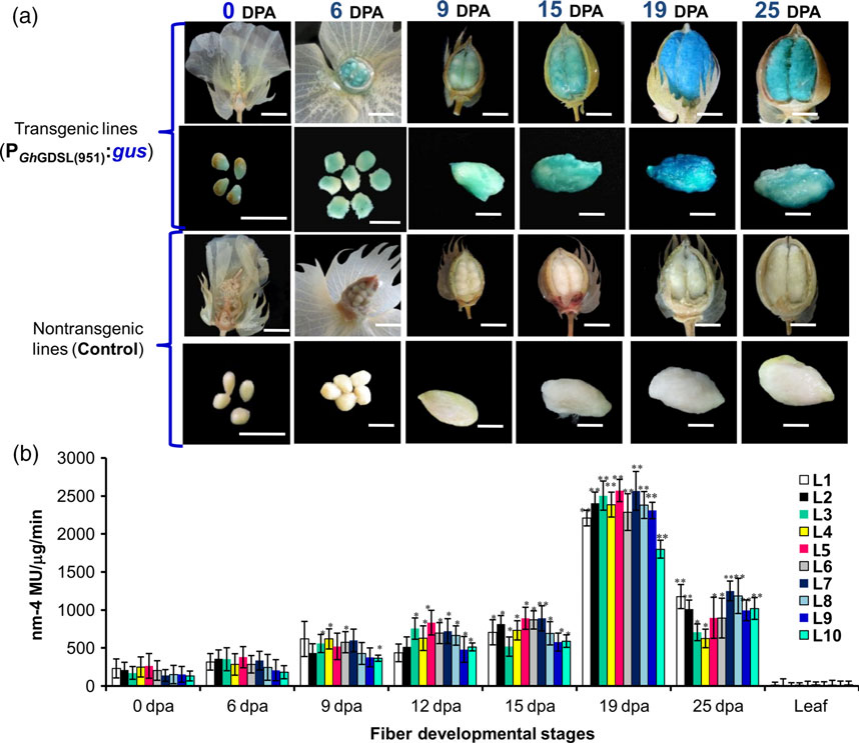
GUS expression analysis in cotton fibre driven by *GhGDSL* promoter. (a) Histochemical GUS staining of P_G__h_ _GDSL_:*gus* expression in T_1_‐transgenic lines and nontransgenic control lines in individual bolls and ovules at the different development stages (0–25 DPA). GUS staining was absent in the nontransgenic cotton bolls and ovules (Scale bar = 0.635 cm). (b) Fluorometric GUS expression analysis of P_G__h_ _GDSL_:*gus* in ten independent cotton transgenic lines: histochemical GUS analysis was performed as described by Jefferson *et al*. (1987). The expression values were normalized using the non‐transgenic control values. The results are the average of three biological replicates of T_1_ lines of the ten independent lines: the asterisk represents statistical *t*‐test between transgenic lines at different DPA compared to 0DPA (*P‐values <0.05 and **P‐values <0.01). The error bars represent ±SE of three independent biological replicates.

We then cloned a 1026‐nucleotide (–951/+75 with respect to the ATG) long P_GhGDSL_ promoter fragment using genome walking (Figures 1c, S4). The presence of the primers and the sequence of an overlapping part of the coding region of *GhGDSL* ensured that the cloned fragment belonged to the upstream promoter region of the same gene identified from the microarray. Genomewide BLAST against the *Gossypium hirsutum* genome (AD) further confirmed that both the promoter and the gene aligned at the same region on chromosome 4 (Table S1). The promoter fragment was fused to the *gus* gene in pBI101 to develop a reporter construct that was mobilized into *Agrobacterium tumefaciens* and used to generate several independent transgenic lines of cotton. We evaluated ten independent T_1_‐transgenic lines for both quantitative and histochemical expression of the *gus* reporter at different stages of fibre development. Histochemical staining of GUS indicated weak expression at 0 DPA as shown by weak blue staining of the ovules (Figure 2a). Expression increased gradually from 0 DPA and reached a maximum at 19 DPA (Figure 2a). The expression was still high even at 25 DPA, but was lower than at 19 DPA. Thin sections of stained ovules at 0, 6 and 19 DPA confirmed the SCW stage‐specific expression of GUS localized within the fibres and the ovule epidermis at 19 DPA. GUS staining was not observed in any other tissues, such as in seedling, root, leaf and bracts (Figure S1). Further, quantitative GUS expression in the ten independent lines, although showing minor differences among lines, was consistently highest at 19 DPA (Figure 2b), coinciding with the pattern of histochemical staining. None of the transgenic lines showed any significant expression in leaves used as controls (Figure 2b). We also developed independent transgenic cotton lines with *gfp* fused to the native *GhGDSL* promoter. There was weak GFP fluorescence at 0 DPA and significantly higher fluorescence at 19 DPA (Figure S2), consistent with the other measures of expression of this gene. Similar to the GUS reporter, no significant GFP expression was observed in any other tissues of the transgenic lines examined (not shown). The analyses in transgenic cotton further validated the SCW stage‐specific expression of *GhGDSL* and indicated that P_GhGDSL_ should be a good candidate to explore the regulatory networks involved in SCW formation during fibre development.

### The *cis*‐regulatory elements controlling the SCW stage‐specific expression of *GhGDSL*

**Figure 3:**
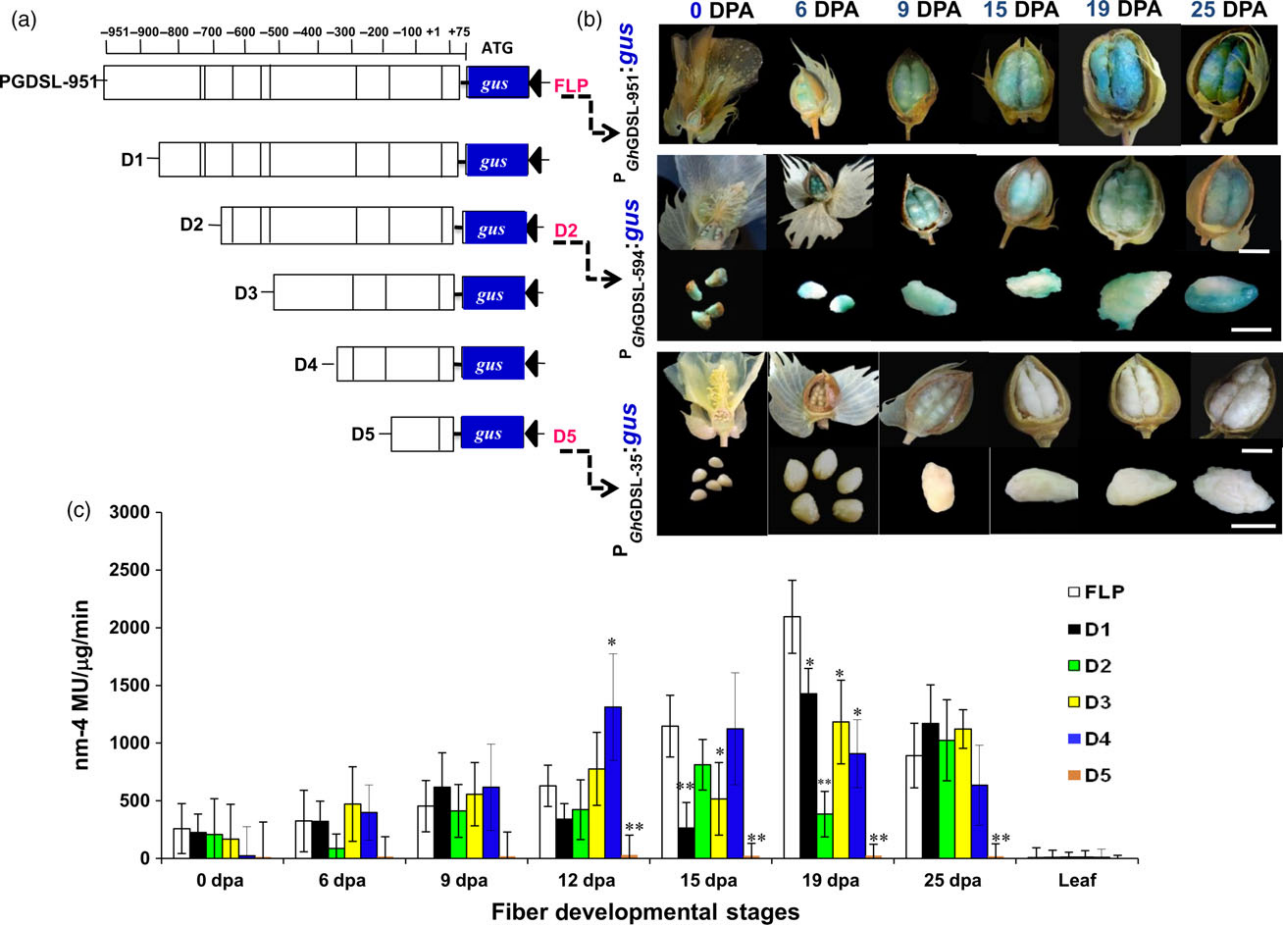
Deletion analysis of the *GhGDSL* promoter. (a) Schematics of P_G__h_ _GDSL_ deletions D1, D2, D3, D4 and D5 along with full‐length promoter. +1 represents TSS. Scale bar represents the length of promoter (–951/+75) from the start codon ATG. (b) Histochemical GUS analysis of the cotton T_1_‐transgenic lines carrying a deletion construct: the upper panel shows the GUS staining of a transgenic cotton flower and bolls of the native promoter reporter (P_G__h_ _GDSL_) during the development stages (0–25 DPA). The middle panel shows the GUS staining of a transgenic cotton flower, boll or ovule carrying P_G__h_ _GDSL__–594_:*gus* (D2) construct. The lower panel indicates that there was no staining in a transgenic cotton flower, boll or ovule with P_G__h_ _GDSL__–35_:*gus* (D5) construct. (c) Fluorometric GUS expression analysis of transgenic cotton lines carrying deletion constructs (D1‐D5). The histochemical GUS analysis was performed as described by Jefferson *et al*. (1987). The expression values are normalized with the control values. The results are the average of three biological replicates for each of 7–10 independent T_1_‐lines: statistical *t*‐test between deletion lines and full‐length promoter (*P‐values <0.05 and **P‐values <0.01). The error bars represent ±SE.

To delineate the *cis*‐regulatory sequences involved in regulating *GhGDSL*, we generated five progressive 5′ deletions of the native full‐length promoter P_GhGDSL–951_ (FLP) that were designated as: P_GhGDSL–788_ (D1), P_GhGDSL–594_ (D2), P_GhGDSL–403_ (D3), P_GhGDSL–221_ (D4) and P_GhGDSL–35_ (D5) (Figure 3a). Each of the promoter deletions was fused to the *gus* reporter gene and the constructs transformed into cotton. GUS activity was analysed in the ovules of 7–10 independent T_1_‐transgenic lines per construct at different fibre development stages. The first deletion construct D1 showed a significant reduction in GUS expression at 15 DPA, but much less at 19 DPA compared to the native P_GhGDSL–951_ (Figure 3b‐c) and was not significantly different to the full‐length promoter at other developmental stages. Interestingly, the second deletion construct D2 showed no significant change in the GUS expression at most developmental stages except for 19 DPA, where expression in the ovules of all the transgenic lines was significantly reduced relative to P_GhGDSL–951_. These results indicated that the *cis*‐regulatory elements, which are present between –788 and –594 nucleotides of the native promoter, regulate 19 DPA stage‐specific expression of *GhGDSL*. The expression of the D3 deletion construct was significantly lower at 15 and 19 DPA as compared to P_GhGDSL–951_. However, its expression at 19 DPA was significantly higher compared to the D2 construct. Similarly, the expression of the D4 construct was significantly lower than P_GhGDSL–951_ but higher than D2 at 19 DPA (Figure 3c). Interestingly, the expression of construct D4 was significantly higher than all the constructs, including the full‐length construct P_GhGDSL–951_ at 12 DPA. The final deletion construct D5 did not show any significant expression at any of the fibre development stages. However, this was expected considering that it contained only minimal promoter sequences. The results for D5 also confirmed that the regulatory region upstream of 110 nucleotide (–35/+75) had all the regulatory sequences necessary for 19 DPA fibre‐specific expression. Furthermore, the expression pattern of P_GhGDSL–951_, D2 and D5 was confirmed by staining the ovules of the transgenic lines at different development stages. The histochemical staining was concordant with the quantitative GUS estimation, confirming the 19 DPA elevated expression of P_GhGDSL–951_ (Figure 3b). D2 showed a reduced staining at all the development stages, especially at 19 DPA confirming that the deletion of the promoter up to –594 results in the loss of 19 DPA‐specific expression of *GhGDSL*. Ovules of the D5 construct did not show GUS staining at any stage.

### MYB1AT motif regulates the 19–25 DPA‐specific expression of *GhGDSL*

**Figure 4:**
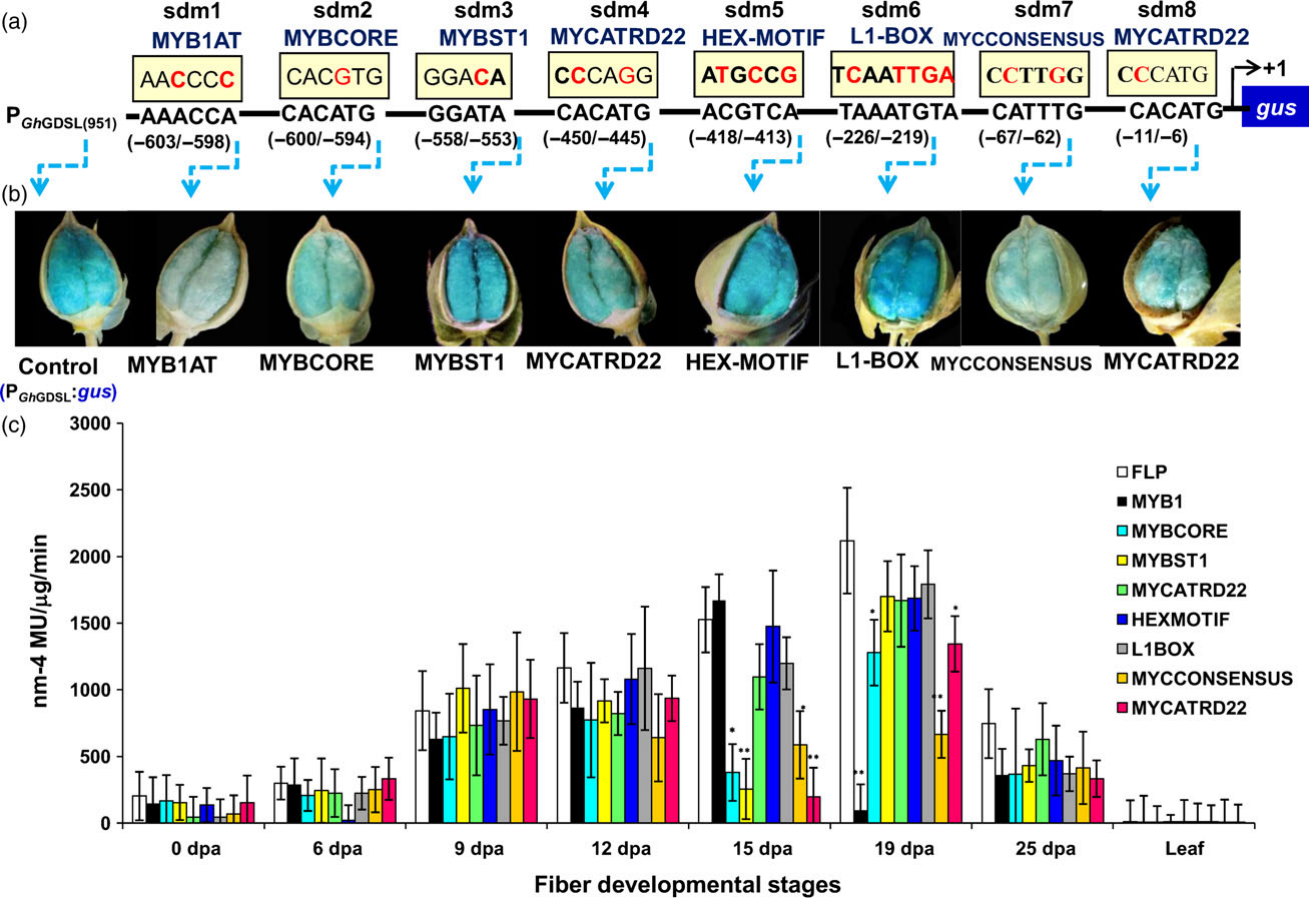
Mutation analysis of *GhGDSL* promoter. (a) Schematics of mutated motifs present in P_G__h_ _GDSL_: the site‐specific mutations in promoter designated as sdm1 (MYB1AT‐AACCCC), sdm2 (MYBCORE‐CACGTG), sdm3 (MYBST1‐GGACA), sdm4 (MYCATRD22‐CCCAGG), sdm5 (HEX‐motif‐ATGCCG), sdm6 (L1BOX‐TCAAATGT), sdm7 (MYCCONSENSUS‐CCTTGG) and sdm8 (MYCATRD22‐CCCATG) are highlighted in red. (b) Histochemical GUS analysis of T_1_‐transgenic cotton lines carrying mutated motifs (sdm1‐sdm8): GUS staining of a transgenic cotton bolls carrying native P_G__h_ _GDSL_ and each of the promoters with the mutated motifs such as MYB1AT, MYBCORE, MYBST1, MYCRD22, HEX‐motif, L1BOX‐TCAAATGT, MYC CONSENSUS and MYCATRD22 at 19 DPA. (c) Fluorometric GUS expression analysis of transgenic cotton lines carrying the promoter with mutated motif constructs (sdm1‐sdm8). The expression values are corrected with the control values. The results are the average of three biological replicates each of 7–10 independent T_1_ lines: statistical *t*‐test between the mutation lines (sdm1‐sdm8) and full‐length promoter (*P‐values <0.05 and **P‐values <0.01). The error bars represent ±SE.

Computational analysis of conserved *cis*‐regulatory elements within P_GhGDSL–951_ using PLACE (www.dna.affrc.go.jp) revealed several conserved motifs in the promoter (Table S2), including some MYB binding motifs such as WAACCA (MYB1AT), CACATG (MYBCORE), GGATA (MYBST1), and bHLH and homeodomain binding motifs such as CACATG (MYCATRD22), TAAATGYA (L1BOX), CATTTG (MYC CONSENSUS) and CACATG (MYCATRD22) being present at –603/–598, –600/–594, –558/–553, –450/–445, –226/–219, –67/–62 and –11/–6 positions, respectively (Figure 1c). MYB transcription factors have been reported to play an important role in cotton fibre development, so we decided to examine their contribution by specifically mutating these MYB and other conserved motifs. Eight mutational constructs, designated as sdm1 to sdm8, were generated by site‐directed mutagenesis of native promoter P_GhGDSL–951_ (Figure 4a). Each of the mutant versions of the promoter was fused to the *gus* gene and transformed in cotton to produce multiple transgenic lines for each construct. At least 7–10 T_1_‐transgenic lines were evaluated for GUS expression in each construct and each of the different development stages of cotton fibre. None of the transgenic lines showed any significant change in quantitative GUS expression from 0 DPA to 12 DPA compared to the native promoter P_GhGDSL–951_ (Figure 4c). However, at 15 DPA, sdm2, sdm3, sdm7 and sdm8 mutations within the MYBCORE, MYBST1, MYCCONSENSUS and MYCATRD22, respectively, showed a significant reduction in GUS activity compared to P_GhGDSL–951_ (Figure 4c). The most striking differences were at the 19 DPA stage, where the sdm1 mutant of the MYB1AT motif almost completely abolished GUS activity. This result indicated that the SCW stage‐specific 19 DPA expression of *GhGDSL* was regulated through the MYB1AT motif. The sdm2, sdm7 and sdm8 mutants also showed a significant reduction in GUS activity compared to P_GhGDSL–951_ at 19 DPA, but their expression was still much higher than the sdm1 construct. In comparison with P_GhGDSL–951_, no major change in the expression of any of the site‐directed mutants was observed at 25 DPA (Figure 4c). Histochemical staining for GUS gave similar results to the quantitative analyses, and only weak staining of cotton bolls was seen for constructs, sdm1 and sdm7 (Figure 4b).

### GhMYB1 interacts with the MYB1AT motif of the *GhGDSL* promoter

**Figure 5:**
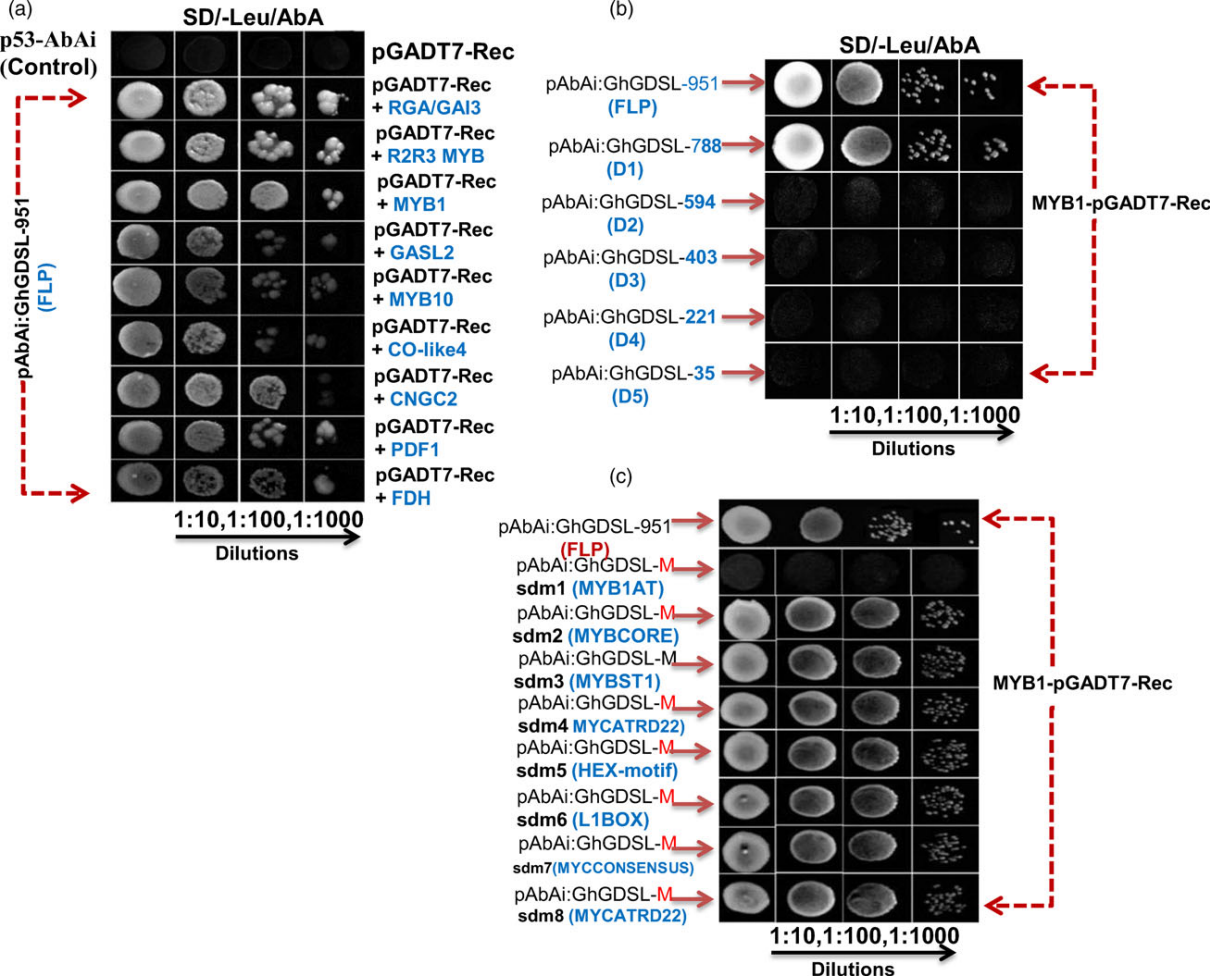
Yeast one‐hybrid interaction of nine TFs and the interaction analysis of GhMYB1 with deletion and mutated motif constructs (sdm1‐sdm8) of the *GhGDSL* promoter. (a) The interaction analysis of nine TFs (RGA/GAI3, R2R3‐MYB, MYB1, GASL2, MYB10, CO‐like4, CNGC2, PDF1 and FDH) on SD/‐Leu/AbA agar plates. Columns are undiluted, 1:10‐, 1:100‐ and 1:1000‐fold dilutions, respectively. SD/‐Leu/AbA represents the selection of positively interacting clones on Leucine drop out medium with AbA antibiotic selection (100 ng/mL). (b) GhMYB1 interaction with P_G__h_ _GDSL_ deletions (D1, D2, D3, D4 and D5) construct. GhMYB1 binds between the −788 and −594 region of P_G__h_ _GDSL_. Yeast colonies were not observed with D3–D5. Columns are undiluted, 1:10‐, 1:100‐ and 1:1000‐fold dilutions, respectively. (c) The interaction analysis of MYB1 with the promoter containing mutated promoter motifs confirmed that GhMYB1 binding with P_G__h_ _GDSL_ at the MYB1AT motif sequence AAACCA. No yeast colony was observed with the mutated motif sequence AAACCA (MYB1AT) of P_G__h_ _GDSL_ but binding was observed for all other mutated motifs.

We next identified putative promoter binding proteins that bind to P_GhGDSL–951_ using the yeast one‐hybrid (Y1H) system. P_GhGDSL–951_ was cloned upstream of the Aureobasidin resistance gene (*AbA* ^*r*^ *)* and used as a bait to screen for potential transcription factors that could activate this reporter in yeast. We used a cDNA library prepared from pools of different stages of fibre development (0, 3, 6, 9, 15, 19 and 25 DPA) fused to the *Gal4* activation domain (AD) for screening. Positive interacting clones were selected on SD medium lacking leucine and containing AbA and the promoter binding proteins identified by sequencing the isolated plasmids from resistant colonies. Nine putative binding proteins (Table S3) that might interact with P_GhGDSL–951_ with a significant affinity, as confirmed by dilution (Figure 5a), were identified. Interestingly, out of the nine, three belong to MYB family, viz. an R2R3‐MYB, MYB10 and MYB1. As our previous deletion and mutational experiments clearly suggested that the MYB1AT motif regulates the 19 DPA stage‐specific expression of *GhGDSL* and *GhMYB1* was here shown to bind to P_GhGDSL–951,_ we confirmed this interaction through further experimentation. All of the promoter deletion constructs and the site‐directed mutagenesis constructs were cloned into the Y1H vectors and their interaction with GhMYB1 fused to *Gal4*‐AD assessed, as for the full‐length promoter. The D1 deletion showed similar binding to GhMYB1 as the native construct (Figure 5b). However, the successive deletions, D2, D3, D4 and D5, did not show any significant binding to GhMYB1 (Figure 5b). This observation clearly indicated that the binding site for GhMYB1 lies between –788 and –594. Mutants sdm2 to sdm8 did not show any significant difference in their binding compared to the native promoter (Figure 5c); however, the mutant of MYB1AT, that is sdm1, showed complete loss of binding, as no growth on selection medium was observed at any dilution tested (Figure 5c). Thus, we confirmed that GhMYB1 does interact with P_GhGDSL–951_ at its MYB1AT binding sites that lies between –788 and –594.

### GhMYB1 is expressed explicitly during SCW synthesis at 19 DPA stage of cotton fibre development

**Figure 6:**
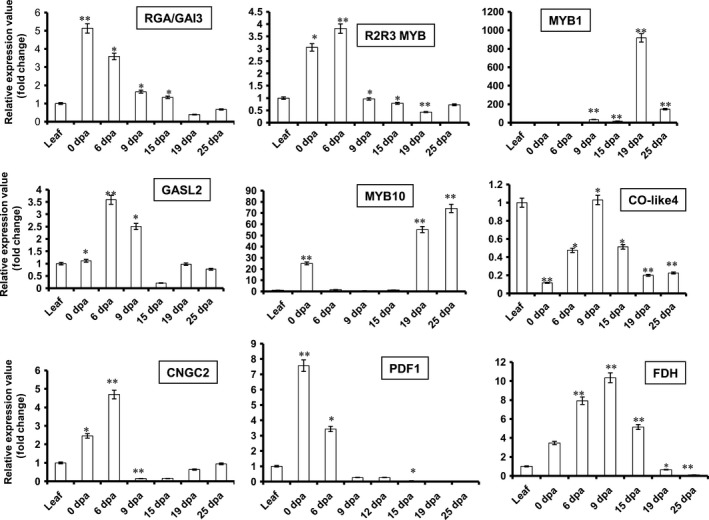
Quantitative real‐time PCR (qRT‐PCR) expression analysis of the nine putative promoter binding proteins identified from Y1H interaction with *GhGDSL* promoter. Expression analysis of RGA/GAI3, R2R3‐MYB, MYB1, GASL2, MYB10, CO‐like4, CNGC2, PDF1 and FDH at different fibre development stages (0–25 DPA): gene expression was normalized with the cotton ubiquitin gene. The error bars represent ±SE of three independent biological replicates. Statistical *t*‐test is represented by the asterisk (*P‐values <0.05 and **P‐values <0.01).

A transcription factor needs to be expressed at a particular development stage in order to regulate a gene specific to that stage. qRT‐PCR expression analysis was carried out for all nine of the identified putative promoter binding proteins using RNA from each of the different stages of fibre development. Many of these putative promoter binding proteins were more highly expressed during fibre development than in leaves (Figure 6). Only *Co‐like4* was expressed at a significantly higher level in leaves than fibre. The transcription factors, such as *RGA/GAI3*,* R2R3‐MYB*,* GASL2*,* CNGC2* and *PDF1* (Figure 6), had high expression during 0 or 6 DPA so are fibre initiation stage‐specific genes. The *FDH* (Figure 6) showed significantly higher expression at 9 DPA indicating that it was elongation stage‐specific. Interestingly, only *GhMYB1* showed a 19 DPA stage‐specific expression (Figure 6) similar to that seen for *GhGDSL* (Figure 1b). *GhMYB1* is clearly co‐expressed with *GhGDSL* at the SCW stage consistent with its likely role in controlling SCW stage‐specific expression of *GhGDSL* and potentially other genes. It is also noteworthy that *MYB10* had high expression at 19 DPA, but its expression appeared to be even higher at 25 DPA (Figure 6).

### GhMYB1 co‐expresses with SCW stage‐specific genes during fibre development

**Figure 7:**
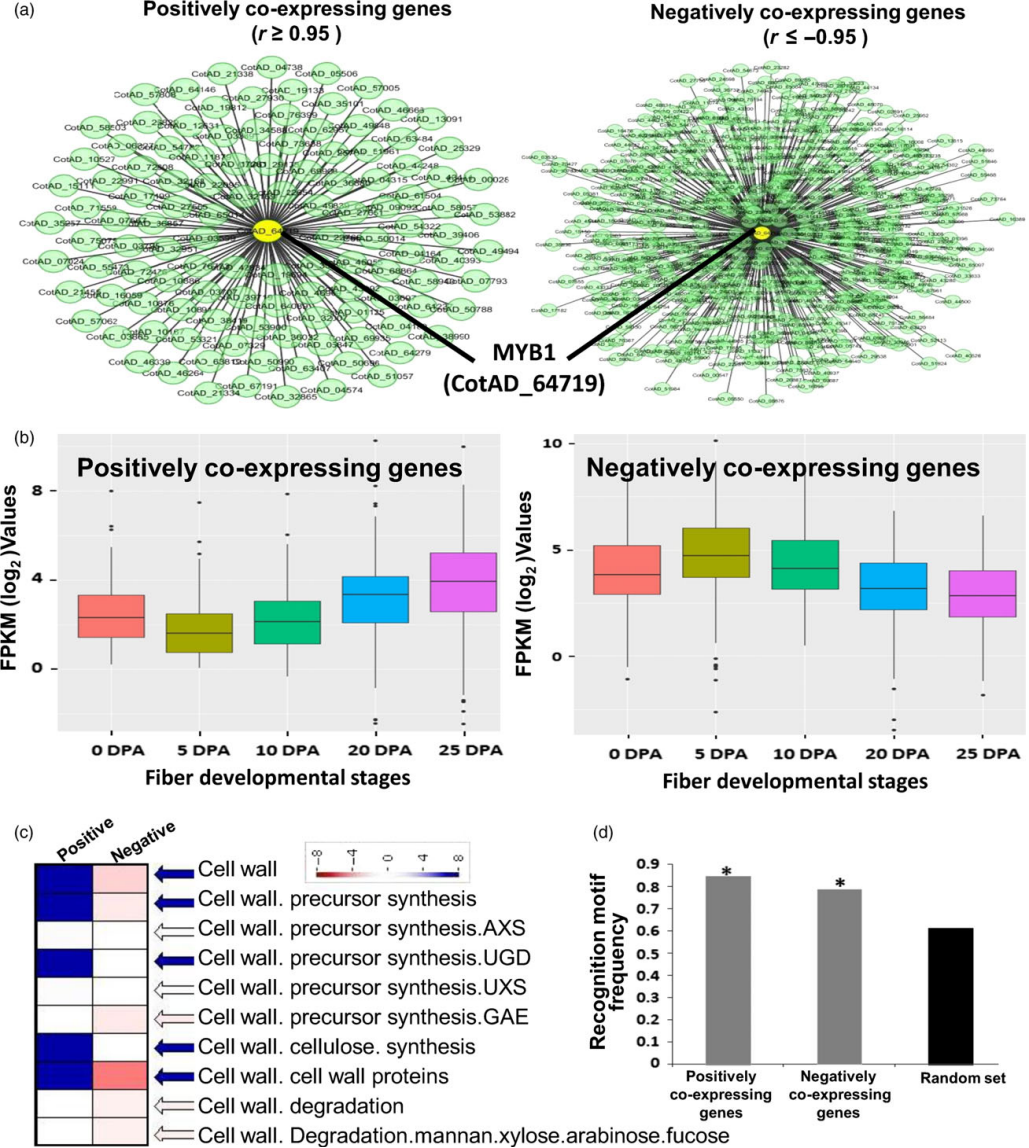
Gene co‐expression network analysis of *GhMYB1*. (a) Unweighted gene co‐expression network of positive and negative partners of *GhMYB1* in different fibre development stages. Circles (nodes) represent transcripts, while the lines (edges) represent a significant transcriptional interaction between *GhMYB1* and the other transcripts. (b) Box plot showing the expression level of the positively and negatively co‐expressing partners of *GhMYB1* at different fibre development stages. (c) MapMan analysis revealed that *GhMYB1* co‐expresses positively with the genes involved in the biosynthesis of the cell wall and its precursors during the fibre SCW stages. (d) Graph showing the frequency of MYB1AT recognition motif (WAACCA) in the promoters of the positively and negatively interacting partners and a randomly selected set of expressed genes. The asterisks represent significant differences (Fisher's exact test, *P* < 0.05).

Genes that are co‐expressed with *GhMYB1* during cotton fibre development from publically available RNA‐seq data of 0, 5, 10, 20 and 25 DPA were identified using the expression correlation network plug‐in available in Cytoscape version 2.8.1 (Shannon *et al*., 2003). A total of 118 positively co‐expressed genes (*r* ≥ 0.95) and 435 negatively co‐expressed genes (*r* ≤ −0.95) were identified (Appendix S1; Figure 7a). The expression levels (based on normalized read counts) of the positively and negatively co‐expressed genes at each fibre developmental stage were assessed (Figure 7b). Genes positively co‐expressed with *GhMYB1* had significantly higher levels of expression during the later stages of fibre development, especially at 25 DPA, while those negatively co‐expressed with *GhMYB1* had lower expression at 25 DPA relative to earlier time points (Figure 7b). Our results therefore suggest that *GhMYB1* may be regulating genes expressed specifically at the SCW stages. We examined the gene ontology classifications of both the positively and negatively co‐expressing genes using MapMan visualization and statistical analysis tools. This revealed that the positively co‐expressing genes were enriched in the cell wall category including some genes involved in the synthesis of cell wall polysaccharides precursors (Figure 7c), although the major biosynthetic gene (like cellulose synthase) for the cell wall polysaccharides was not indicated (Appendix S1). The positively expressing genes were also enriched for genes involved in carbohydrate metabolism and secondary metabolism of isoprenoid and phenylpropanoid compounds (although again not the major pathway enzymes) that are minor components of cell wall (Figure S3). The presence of MYB1AT binding motif (Figure 7d) in the promoters (1000 nucleotide upstream of TSS) of the positively co‐expressed, negatively co‐expressed, and 150 randomly selected genes, as the control, was examined. The control data set showed a random frequency of promoters containing MYB1 motifs at 0.61, while positively and negatively regulated genes showed frequencies of 0.84 and 0.78, respectively, which were significantly higher than expected by chance. These results suggest that GhMYB1 could be up‐regulating the positively co‐expressed genes by binding to the MYB1AT motifs in their promoters. GhMYB1 might also be interacting with the negatively co‐expressed genes, although further detailed experimentations would be needed to confirm a repressive role for GhMYB1 during fibre development.

## Discussion

SCW biosynthesis is a critical stage of fibre development, characterized by rapid deposition of cellulose and is known to influence fibre quality traits such as length, strength and micronaire in cotton. Despite the plethora of information available about cotton fibre initiation and its regulation, very little is known about the transcriptional regulation of SCW biosynthesis in cotton. To decipher the transcription regulation of the SCW, we cloned and characterized the SCW biosynthesis stage‐specific P_GhGDSL_ in *G. hirsutum*. P_GhGDSL_ was selected on the basis of our previous work (Nigam *et al*., 2013) on expression profiling of five genotypes of cotton at six different development stages, where *GhGDSL* was found to be one of the significantly up‐regulated genes during fibre SCW deposition stages (19 DPA and 25 DPA; Figure 1a). Quantitative RT‐PCR confirmed that *GhGDSL* is expressed at very high level during SCW stage (Figure 1b). GDSL lipase is known to play important roles in the cellulose deposition and the synthesis of the secondary cell wall in other plant species and also alters pectin composition by the esterification of pectin compounds in *Arabidopsis* (Bischoff *et al*., 2010).GDSL lipase has been found to be involved in several plant processes, but its exact role in cotton fibre development is still elusive. The fibre SCW stage‐specific expression of *GhGDSL* does implicate a possible role for this enzyme in SCW biosynthesis. The *gus* gene driven by P_GhGDSL–951_ in transgenic cotton lines showed a very high level of expression of GUS from 19 DPA and confirmed its SCW stage‐specific expression during cotton fibre development. The expression of P_GhGDSL–951_ began around 6 DPA and reached its maximum level at 19 DPA. No background expression of P_GhGDSL–951_ in any other floral part, or other tissues was observed in any of the transgenic lines, indicating that P_GhGDSL_ is strictly fibre‐specific. Fibre‐specific expression (mostly initiation and elongation stage‐specific) of other promoters, such as *GhGlcAT1* (Wu *et al*., 2007), *FSltp4* (Delaney *et al*., 2007), *GhACTIN1* (Li *et al*., 2005a), *GhLTP6*,* GaMYB2* and *GhMYB109* (Hsu *et al*., 1999; Pu *et al*., 2008; Wang *et al*., 2004), *GhRING1* (Ho *et al*., 2010) *GaRDL1*,* GhTUB1* and *GhMYB25* (Li *et al*., 2002; Machado *et al*., 2009), has been reported previously. However, to the best of our knowledge, there are no other SCW‐specific promoters characterized in any detail so far in cotton. SCW stage‐specific expression of genes, such as *GhRLK1* (Li *et al*., 2005b), *GhKNL1* (Gong *et al*., 2014) and *GhCesA* (Fagard *et al*., 2000; Kim and Triplett, 2001), has been reported, but not studied in the way described here in transgenic cotton plants. The 5′ deletion experiments revealed that the major *cis*‐regulatory element that control 19 DPA‐specific expression of *GhGDSL* lie within the 194‐bp regions between –788 and –594 nucleotide upstream of the transcription start site, as deletion D2 completely abolished the expression of *GhGDSL* at 19 DPA (Figure 3b‐c). The deletion constructs D3 and D4 showed higher expression than D2, indicating that the absence of these upstream elements allows other downstream regulatory elements to become accessible, although these still need to be characterized in detail. The upstream regulator(s) also seem to be essential for the control of expression at 15 DPA and 12 DPA, because the expression of D2 is lower than the full‐length promoter at both these stages. At 25 DPA, however, the regulators within the 194 nucleotide region do not appear to be essential, as the expression of D2 is almost identical to that of the full‐length control at 25 DPA. The deletion experiments suggest that the 194 nucleotide region controls the expression of *GhGDSL* from elongation through to the SCW biosynthesis stage.

MYB transcription factors are known to play a major role during the different stages of cotton fibre development. *GhMYB109*, for example, is expressed during fibre elongation in *G. hirsutum* (Pu *et al*., 2008), while *GhMYB25*,* GaMYB2* and *GhMYB25 like* have been identified to play key roles during initiation and early elongation stages (Deng *et al*., 2011; Walford *et al*., 2011; Wang *et al*., 2004). In the model plant *Arabidopsis*, it has been reported that MYBs play a role in trichome initiation, pattering, trichrome cell fate determination and are also involved in cell wall thickening (Oppenheimer *et al*., 1991; Zhong *et al*., 2008). Thus, we decided to examine the presence of conserved *cis*‐regulatory elements in the promoter of *GhGDSL*, particularly of MYB TF binding sites. P_GhGDSL_ had eight potential *cis*‐acting promoter elements, ranging between 11 and 603 nucleotides upstream of the TSS. The independent mutagenesis of each of these motifs showed that the mutation of MYB1AT at 603 nucleotide led to a significant loss of expression of P_GhGDSL_ at 15 and 19 DPA (Figure 4c). A mutation in the MYBCORE, that overlaps the MYB1AT elements, also resulted in a significant loss of expression of P_GhGDSL_ at 15 and to a lesser extent at 19 DPA. However, the MYB1AT mutation seemed to completely abolish the expression of the promoter at 19 DPA, indicating that it is the major element responsible for the regulation of *GhGDSL* at that stages (Figure 4c). The MYB1AT element lies within the 194 nucleotide regulatory region identified by 5′ deletion. Thus, these observations support the regulation of P_GhGDSL_ through the MYB1AT motif. The MYB1 recognition site was first reported in the promoter region of the ABA and drought responsive *rd22* gene (Abe *et al*., 2003), and it was found to function as a *cis*‐acting element that regulates the expression of the *rd22* gene. The fact that the MYB1AT motif regulates P_GhGDSL_ led us to identify the transcription factors that bind to P_GhGDSL_. Y1H screening of a fibre‐specific expression library identified nine putative promoter binding proteins (Table S3; Figure 6). Six of these have their binding sites conserved within the P_GhGDSL_ (Table S4) region, indicating that they interact specifically with it. Interestingly, GhMYB1 was identified as binding to P_GhGDSL_ with a high affinity. Besides GhMYB1, two other MYB transcription factors, R2R3‐MYB and MYB10, also bound P_GhGDSL_ with a high affinity, but the site(s) to which they bind need to be characterized further. We also identified other proteins, such as the repressor of gibberellic acid (RGA/GAI3) like factor, gibberellic acid stimulated like 2 (GASL2), cyclic nucleotide gated channel isoform 2 (CNGC2), the *G. hirsutum* protodermal factor 1 (GhPDF1), constans‐like 4 (GhCO‐like 4) and Fiddlehead‐like protein (FDH). Some of these, such as GhPDF1, are involved in cotton fibre initiation and elongation, while RGA/GAI3 like factors may play some role in gibberellic acid (GA) signalling in cotton fibre elongation, and GASL2 is involved in fibre elongation in response to GA signalling (Table S3).

As 5′ deletions and mutagenesis clearly indicated that MYB1AT is important in the 19 DPA‐specific expressions of P_GhGDSL_ and Y1H also showed that GhMYB1 bound to this promoter, we further examined whether GhMYB1 interacts specifically with the MYB1AT element using Y1H. GhMYB1 failed to interact with any deletion beyond P_GhGDSL–594_, indicating that its binding site lies upstream of −594 (Figure 5b). Further, only the mutation in MYB1AT (sdm1) −603 relative to TSS resulted in a complete loss of interaction between GhMYB1 and P_GhGDSL_ in the yeast (Figure 5c). Thus, both results strongly suggest that the regulation of P_GhGDSL_ by GhMYB1 is due to its interaction with MYB1AT in the promoter. Further, unlike the other TFs that bound to P_GhGDSL_, *GhMYB1* was expressed maximally from 19 DPA (Figure 6), suggesting that it not only regulates *GhGDSL*, but is potentially involved in the regulation of other genes expressed during the SCW stage of fibre development. Furthermore, we identified a suit of genes that are both positively and negatively co‐expressed with *GhMYB1*. Interestingly, the genes positively co‐expressed with *GhMYB1* are generally expressed at significantly higher levels during the later stages of fibre development (20 DPA and 25 DPA; Figure 7b), while the genes that are negatively co‐expressed with *GhMYB1* were more highly expressed at the initiation or elongation stages (Figure 7b). Some of the positively co‐expressed genes belong to the cell wall precursors functional categories (Figures 7c, S3), including a UDP glucose dehydrogenase that is required for pectin and hemicellulose synthesis and an alpha‐1,4‐glucan–protein synthase (UDP glucose forming) (UPTG, EC 2.4.1.112) that may have a role in cellulose synthesis and genes of the isoprenoid and phenylpropanoids pathways. Phenylpropanoids are an important but minor component of the secondary cell wall, and mature cotton fibre contains approximately 94% of cellulose and traces of what may be lignin like polymers (Fan *et al*., 2009). *GhMYB1* is expressed precisely during the later stages of fibre development overlapping with SCW deposition, but the lack of the core cell wall polysaccharide biogenesis genes such as the SCW CesA, KORRIGAN and FLAs (Fasciclin‐like arabinogalactan proteins), etc., that are characteristics of most SCWs, suggests that *GhMYB1* is regulating a specialized subcomponent of the SCW involving secondary metabolite synthesis, and stress hormone signalling‐related gene network. In accordance with the expression of positively and negatively co‐expressed genes with *GhMYB1*, we also identified that the promoters of both the groups of genes have significantly higher representation of the MYB1AT motif in their promoters (Figure 7d). A high occurrence of an MYB1AT motif in the positively co‐expressing genes with *GhMYB1* implies that MYB1 is involved in the regulation of the genes that are similar to those we established for *GhGDSL*. However, there are a significant number of the negatively co‐expressing genes (435 genes), and interestingly, they also have a very high occurrence (0.78) of the MYB1AT motif. Thus, our results indicated that MYB1AT might be involved in the negative regulation of fibre initiation‐specific genes in the later stages of fibre development. However, the negative role of *GhMYB1* in the regulation of initiation‐specific genes during the later stages of fibre development is just a speculation as at the moment we do not have any experimental evidence to support it.

In conclusion, the current study identified a SCW stage‐specific promoter P_GhGDSL_, which is activated during the later stages of fibre development. This promoter could be used for a variety of biotechnological applications in cotton. Furthermore, our study also indicates that a suit of other SCW biosynthesis stage‐specific genes are also regulated by *GhMYB1*, making it a target gene for the improvement of fibre quality traits in cotton varieties.

## Experimental procedures

#### Plant materials

The cotton plants (*G. hirsutum* L. acc JKC725 a superior fibre quality genotype and Coker 312 genetic standard of *G. hirsutum*) were grown in a glasshouse at CSIR‐National Botanical Research Institute (NBRI), Lucknow, India. DNA and RNA were extracted from the cotton plants grown under standard field conditions. On the day of anthesis (0 DPA), cotton flowers were tagged and bolls were harvested for RNA isolation at 0, 6, 9, 12, 19 and 25 DPA stages. The harvested cotton tissues were quickly stored in ice, and the fibre from ovule was dissected followed by grounding in liquid nitrogen. An entire ovule was taken at 0 DPA for RNA isolation. Root and leaf samples were collected from 20‐day‐old seedlings.

##### Microarray and Real‐time PCR (RT‐PCR) analysis

The details are provided in the Appendix S2.

##### Genome walking and sequence analysis

The details are provided in Appendix S2.

##### Histochemical GUS staining and GUS assay

The details are provided in Appendix S2.

##### Microtomy and light microscopy

The details are provided in Appendix S2.

##### Fluorescence of GFP expressed in cotton fibre

The details are provided in Appendix S2.

#### Deletion analysis of promoter activity in transgenic cotton fibre

The sequence of full‐length P_GhGDSL_ was used as a template for deletions. A forward primer with *SalI* and a reverse primer with *BamHI* restriction enzyme site were used to generate 5′ deletion constructs by PCR. The amplified PCR fragments were ligated into the plasmid pBI101 (Clontech, USA) with T4 DNA ligase (NEB) and the deletions constructs pBI‐P_GhGDSL‐D_:*gus* were named as D1 (pBI‐P_GhGDSL–788_:*gus*), D2 (pBI‐P_GhGDSL–594_: *gus*), D3 (pBI‐P_GhGDSL–403_:*gus*), D4 (pBI‐P_GhGDSL–221_:*gus*) and D5 (pBI‐P_GhGDSL–35_:*gus*). The transformation of the constructs in cotton was carried out using the embryo transformation method (Kumar *et al*., 2013). A forward primer 5′‐ATGATTGAACAAGATGGATTGCACG‐3′ (Npt–2 forward primer) and a reverse primer 5′‐TCAGAAGAACTCGT CAAGAAGGC‐3′ (Npt–2 reverse primer) were used to confirm the positive transgenic cotton lines by PCR (Figure S5).

#### Site‐directed mutagenesis (SDM) analysis of promoter activity in transgenic cotton

GeneArt^®^ Site‐Directed Mutagenesis System (Cat No. A13282, Invitrogen) was used to introduce total eight mutations in the motifs (MYB1AT, MYBCORE, MYBST1, MYC AT RD22, HEXMOTIF, L1BOX, MYC CONSENSUS and MYCATRD22) of P_GhGDSL_ at loci –603/–598, –600/–594, –558/–553, –450/–445, –418/–413, –226/–219, –67/–62 and –11/–6. The mutagenic forward and reverse primers (Table S5) were synthesized (Applied Biosystems, Fostercity, CA, USA) following the instructions (Invitrogen, Carlsbad CA, USA) and used to generate the mutagenic construct by PCR. The mutation constructs (sdm) were confirmed by a 96 capillary automated sequencer (ABI 3730 DNA Analyzer) using a vector specific T3 forward primer (5′‐AATTAACCCTCAC TAAAGGG‐3′) and a T7 reverse primer (5′‐GTAATACGACTCAC TATAGGGC‐3′). The confirmed plasmids were digested with restriction enzymes (*SalI*‐*BamHI*) and ligated into the plasmid pBI101 (Clontech, Terra Bella, CA, USA) using T4 DNA ligase (NEB). The mutagenic constructs were named as sdm1 to sdm8. All the constructs were transformed in cotton by embryo transformation method (Kumar *et al*., 2013). The forward primer 5′‐ATGATTGAACAAGATGGATTGCACG‐3′ (Npt–2 forward primer) and reverse primer 5′‐TCAGAAGAACTCGTCAAGAAGGC‐3′ (Npt–2 reverse primer) were used to confirm the positive transgenic cotton lines by PCR (Figure S5).

##### Transformation of cotton

The details are provided in Appendix S2.

##### Yeast one‐hybrid assay (Gold Y1H)

The details are provided in Appendix S2.

##### Gene co‐expression network analysis of MYB1AT

The details are provided in Appendix S2.

#### Pathway analysis of positively and negatively interacting partners of MYB

The metabolic pathways or cellular processes of positively and negatively interacting genes were analysed by MapMan software version 3.5.1 (http://gabi.rzpd.de/projects/MapMan/). An average statistical test followed by the Benjamini–Hochberg was used to identify the functional categories (BINSs, subBINs) enriched in these genes. This software was used to visualize the amplitudes of the changes in the expression of individual genes belonging to the metabolic pathways or cellular processes.

## Conflicts of interest

The authors declare no conflicts of interest.

## Supporting information


**Figure S1** GUS expression analysis.
**Figure S2** GFP expression analysis in cotton fiber driven by *GhGDSL* promoter.
**Figure S3** MapMan analysis of the positive and negative interacting partners of MYBs.
**Figure S4** Isolation of *GhGDSL* promoter by genome walking.
**Figure S5** Screening the transgenic lines by PCR.
**Figure S6** Estimation of cellulose content as described by Updegraff (1969).
**Figure S7** Phylogenetic analysis of GDSL (gene id CotAD_74480) with *Arabidopsis*.
**Table S1** BLAST result of P_GhGDSL_ and *GhGDSL* gene.
**Table S2** Putative motifs identified in P_GhGDSL._
**Table S3** Putative promoter binding proteins identified by Y1H system.
**Table S4** Motif search for the TFs identified by Y1H.
**Table S5** Primers used in this study.

**Appendix S1** Positively and negatively co‐expressed genes.

**Appendix S2** Supplementary materials and methods.
