# Supplementary material for: GhMYB1 regulates SCW stage‐specific expression of the GhGDSL promoter in the fibres of Gossypium hirsutum L
Source: Plant Biotechnol J. 2017 Mar 23;15(9):1163–74. doi: 10.1111/pbi.12706 (PMC5552479; doi:10.1111/pbi.12706)
Supplement: Supplementary file 1 — Figure S1 GUS expression analysis. Figure S2 GFP expression analysis in cotton fiber driven by GhGDSL promoter. Figure S3 MapMan analysis of the positive and negative interacting partners of MYBs. Figure S4 Isolation of GhGDSL promoter by genome walking. Figure S5 Screening the transgenic lines by PCR. Figure S6 Estimation of cellulose content as described by Updegraff (1969). Figure S7 Phylogenetic analysis of GDSL (gene id CotAD_74480) with Arabidopsis. Table S1 BLAST result of PGhGDSL and GhGDSL gene. Table S2 Putative motifs identified in PGhGDSL. Table S3 Putative promoter binding proteins identified by Y1H system. Table S4 Motif search for the TFs identified by Y1H. Table S5 Primers used in this study. [file PBI-15-1163-s002.ppt]

## Slide 1
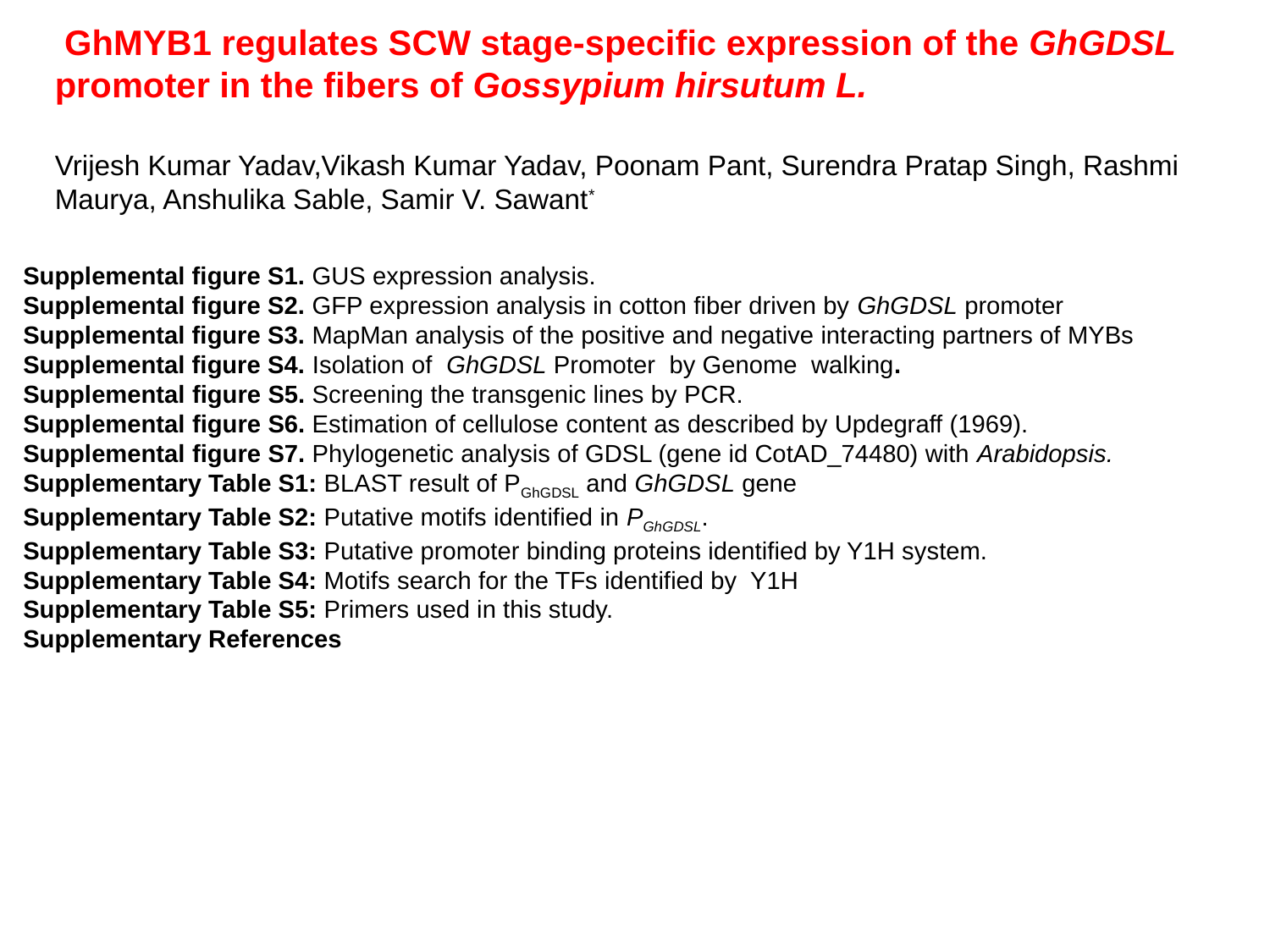

GhMYB1 regulates SCW stage-specific expression of the GhGDSL promoter in the fibers of Gossypium hirsutum L.
Vrijesh Kumar Yadav,Vikash Kumar Yadav, Poonam Pant, Surendra Pratap Singh, Rashmi Maurya, Anshulika Sable, Samir V. Sawant*
Supplemental figure S1. GUS expression analysis.
Supplemental figure S2. GFP expression analysis in cotton fiber driven by GhGDSL promoter
Supplemental figure S3. MapMan analysis of the positive and negative interacting partners of MYBs
Supplemental figure S4. Isolation of GhGDSL Promoter by Genome walking.
Supplemental figure S5. Screening the transgenic lines by PCR.
Supplemental figure S6. Estimation of cellulose content as described by Updegraff (1969).
Supplemental figure S7. Phylogenetic analysis of GDSL (gene id CotAD_74480) with Arabidopsis.
Supplementary Table S1: BLAST result of PGhGDSL and GhGDSL gene
Supplementary Table S2: Putative motifs identified in PGhGDSL.
Supplementary Table S3: Putative promoter binding proteins identified by Y1H system.
Supplementary Table S4: Motifs search for the TFs identified by Y1H
Supplementary Table S5: Primers used in this study.
Supplementary References

## Slide 2
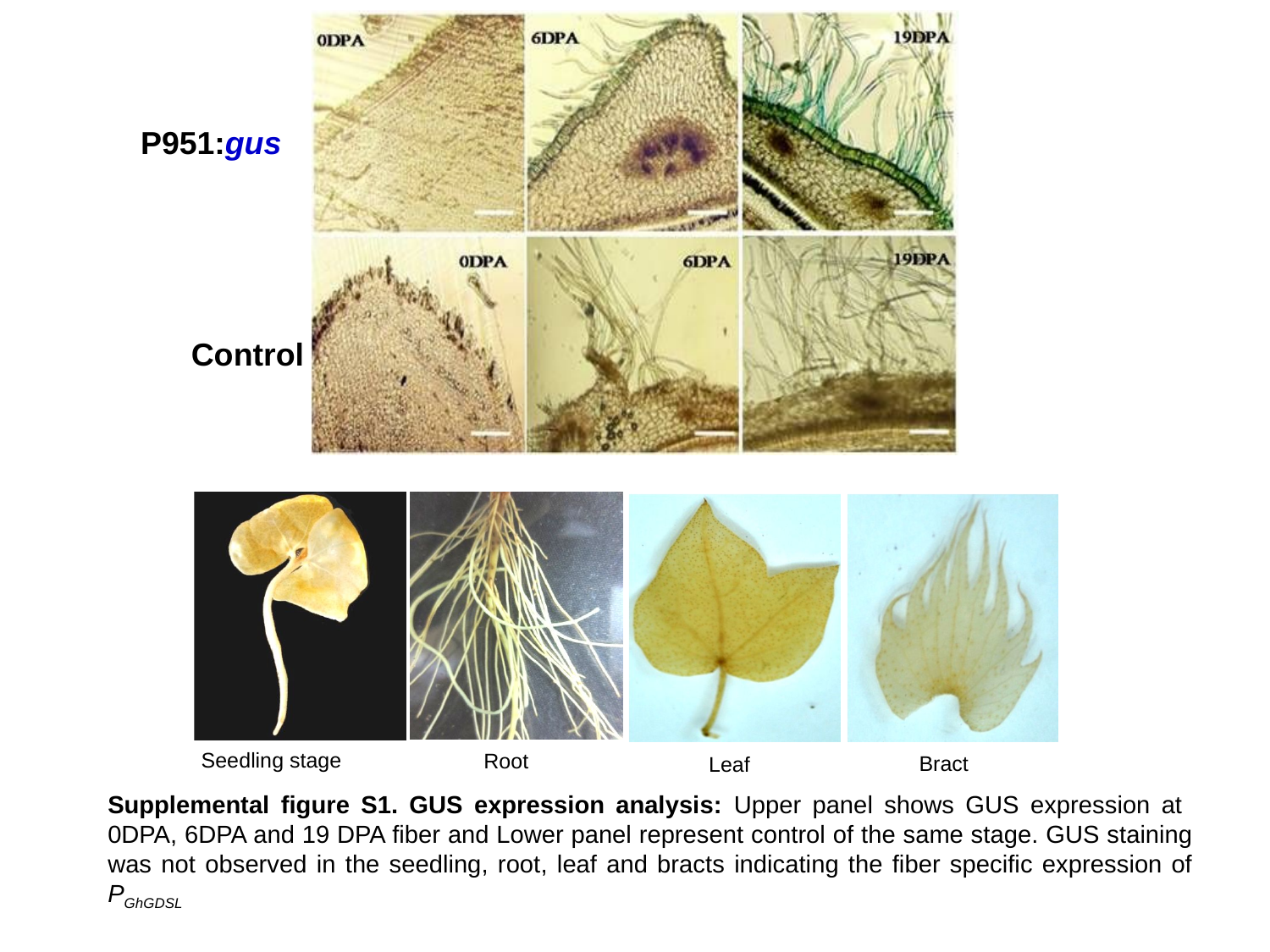

P951:gus
Control
Seedling stage
Root
Bract
Leaf
Supplemental figure S1. GUS expression analysis: Upper panel shows GUS expression at 0DPA, 6DPA and 19 DPA fiber and Lower panel represent control of the same stage. GUS staining was not observed in the seedling, root, leaf and bracts indicating the fiber specific expression of PGhGDSL

## Slide 3
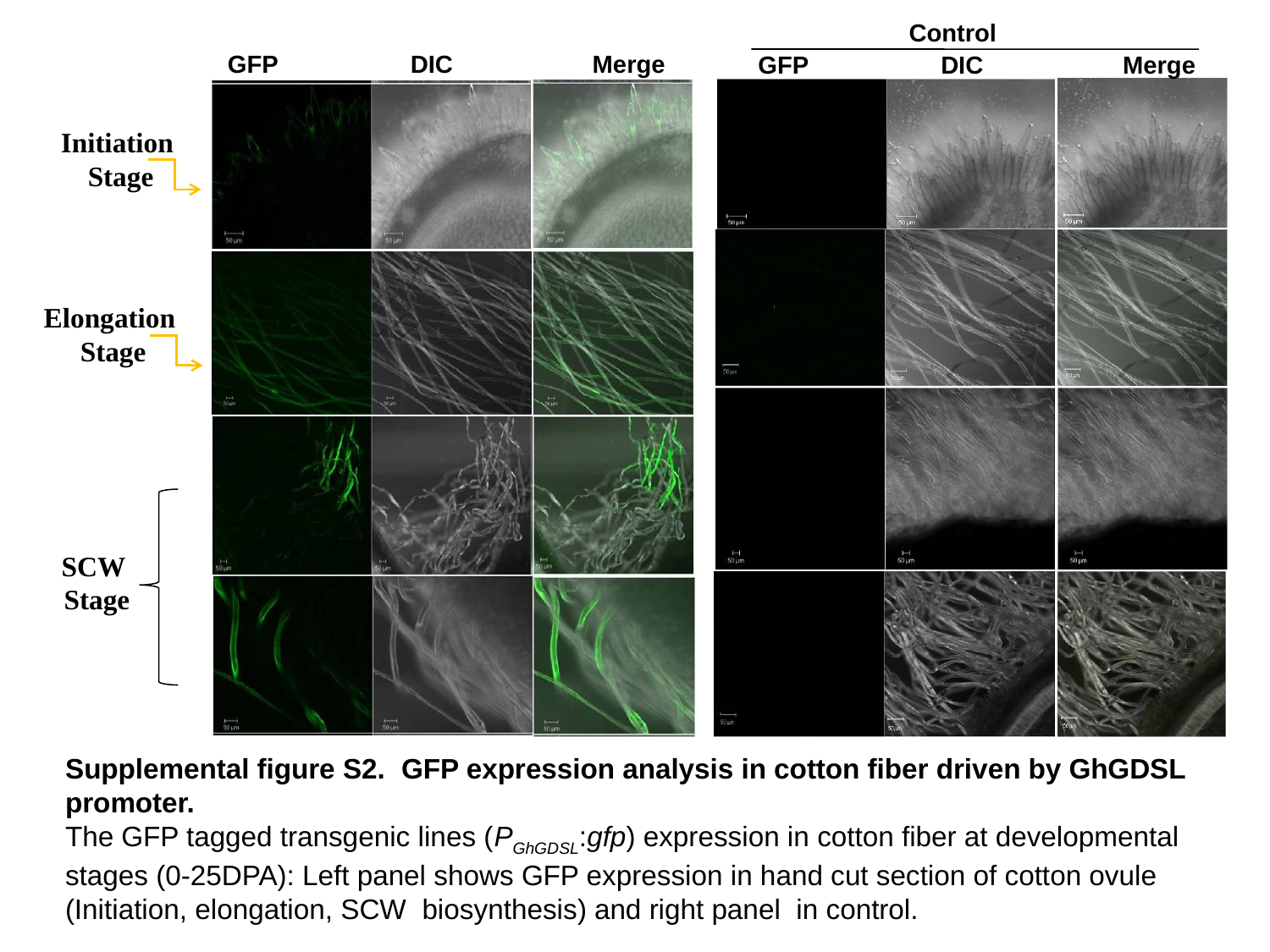

Control
GFP DIC Merge
GFP DIC Merge
Initiation
Stage
Elongation
Stage
SCW
Stage
Supplemental figure S2. GFP expression analysis in cotton fiber driven by GhGDSL promoter.
The GFP tagged transgenic lines (PGhGDSL:gfp) expression in cotton fiber at developmental stages (0-25DPA): Left panel shows GFP expression in hand cut section of cotton ovule (Initiation, elongation, SCW biosynthesis) and right panel in control.

## Slide 4
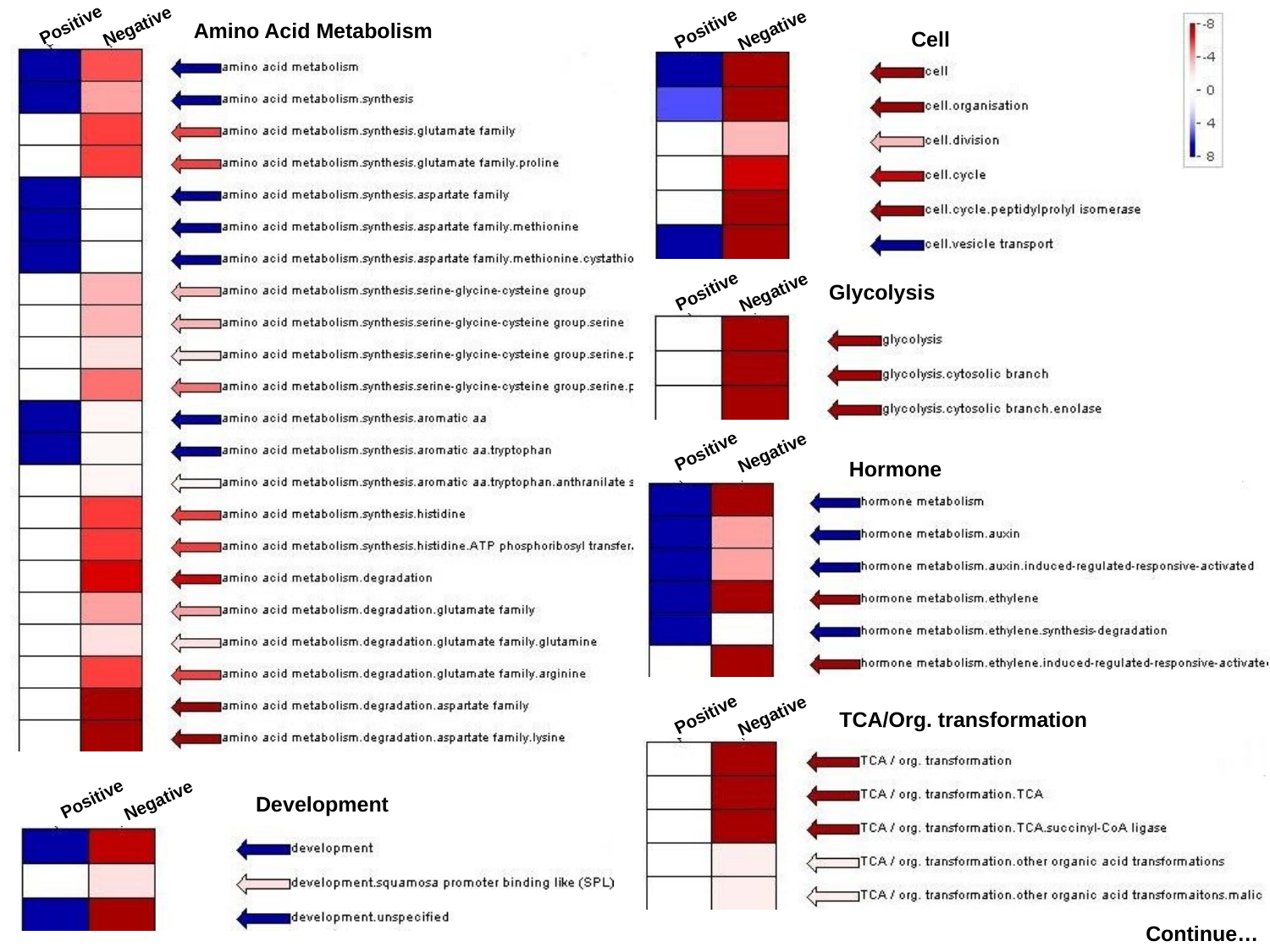

Positive
Negative
Positive
Amino Acid Metabolism
Negative
Cell
Positive
Glycolysis
Negative
Positive
Negative
Hormone
Positive
Negative
TCA/Org. transformation
Positive
Negative
Development
Continue…

## Slide 5
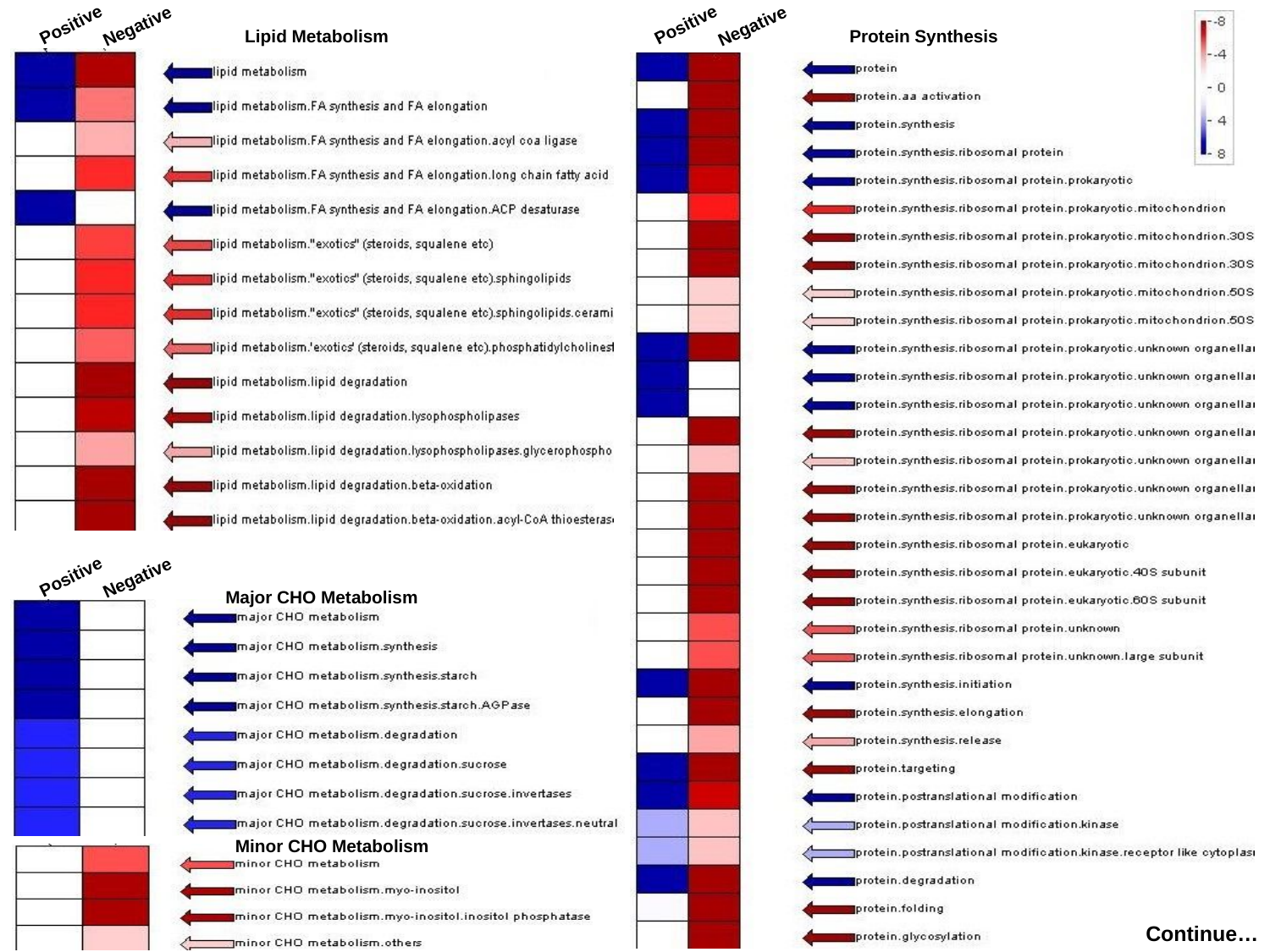

Positive
Positive
Negative
Negative
Lipid Metabolism
Protein Synthesis
Positive
Negative
Major CHO Metabolism
Minor CHO Metabolism
Continue…

## Slide 6
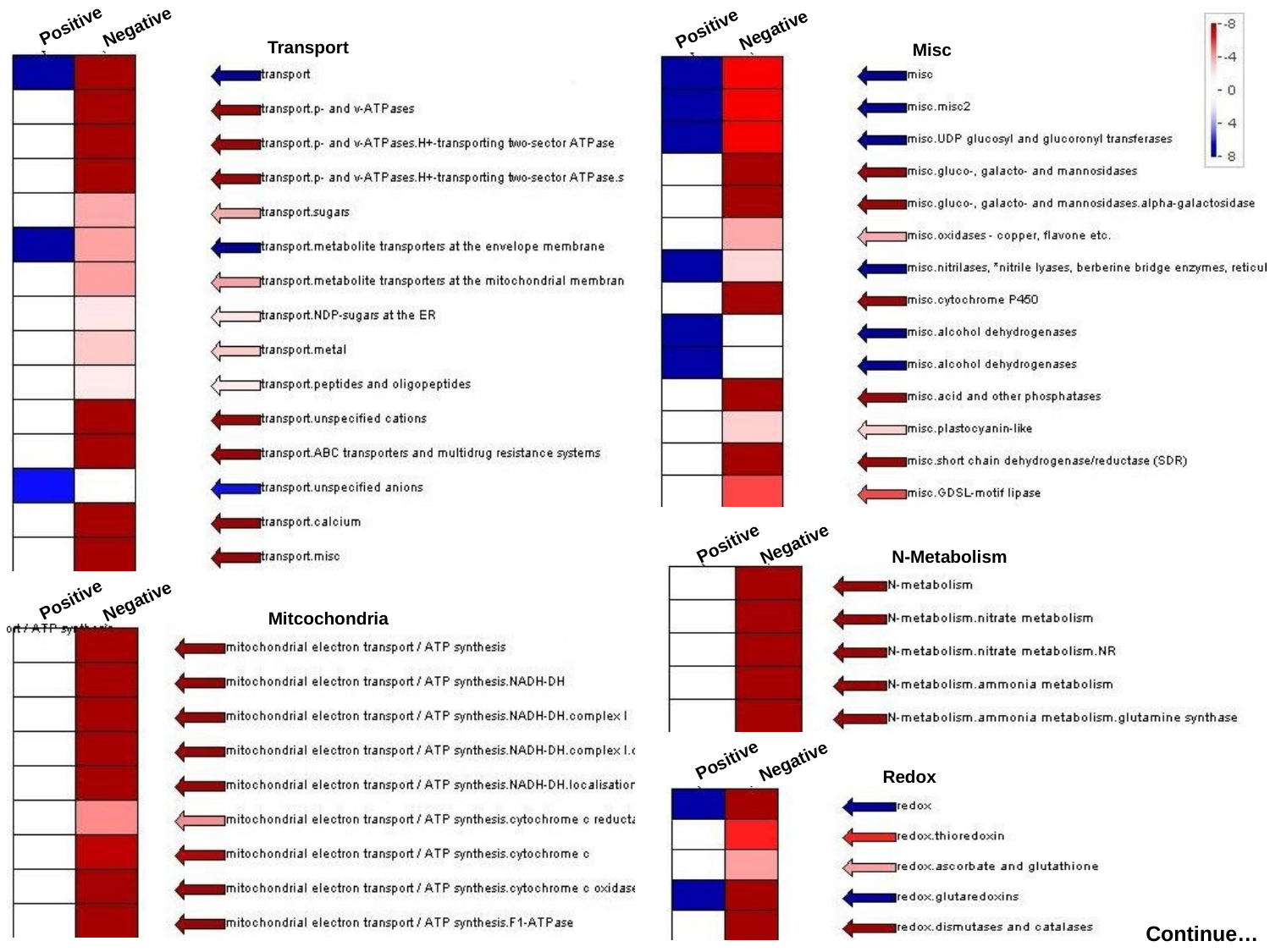

Positive
Negative
Positive
Negative
Transport
Misc
Positive
Negative
N-Metabolism
Positive
Negative
Mitcochondria
Positive
Negative
Redox
Continue…

## Slide 7
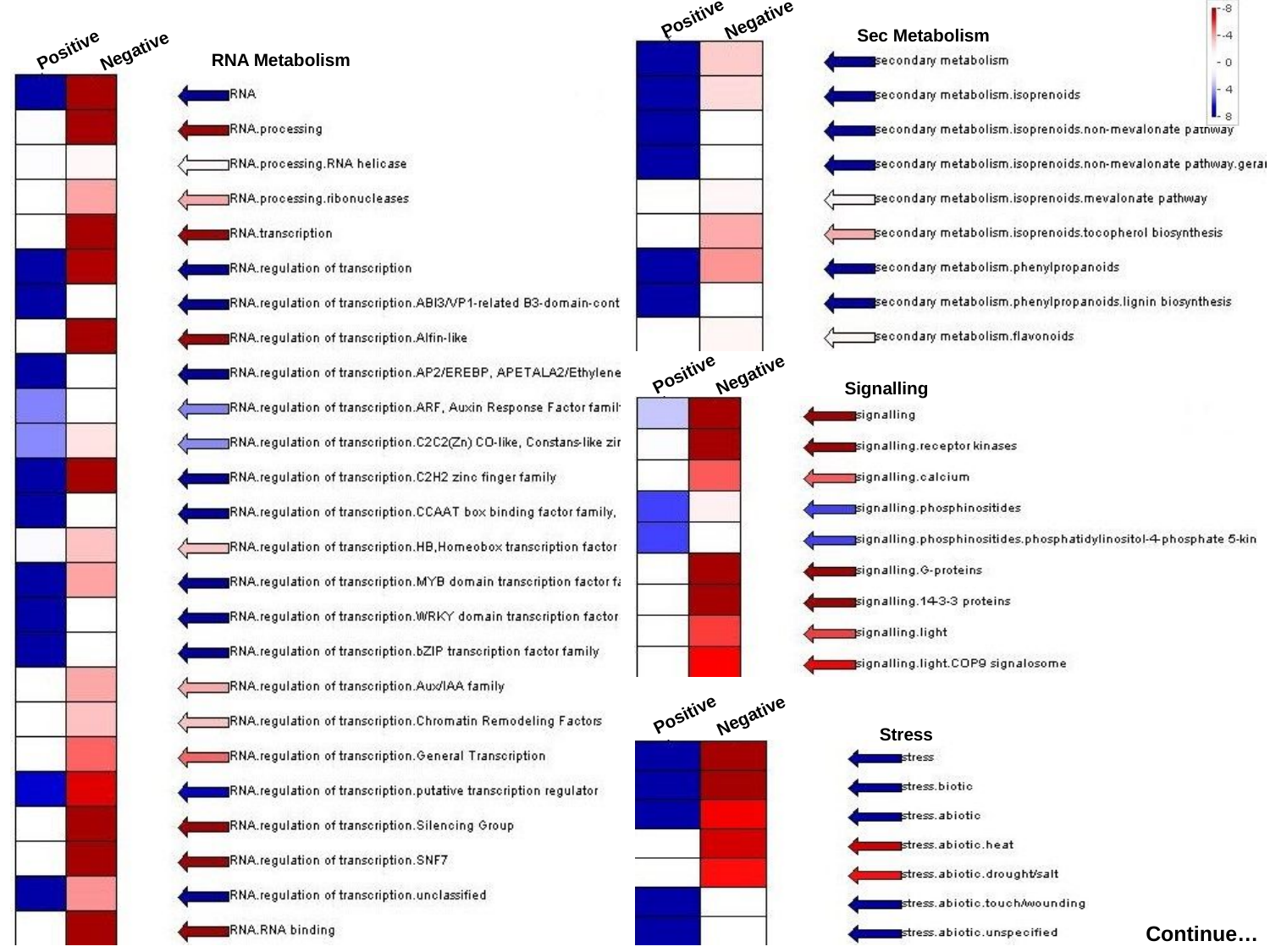

Positive
Negative
Sec Metabolism
Positive
Negative
RNA Metabolism
Positive
Negative
Signalling
Positive
Negative
Stress
Continue…

## Slide 8
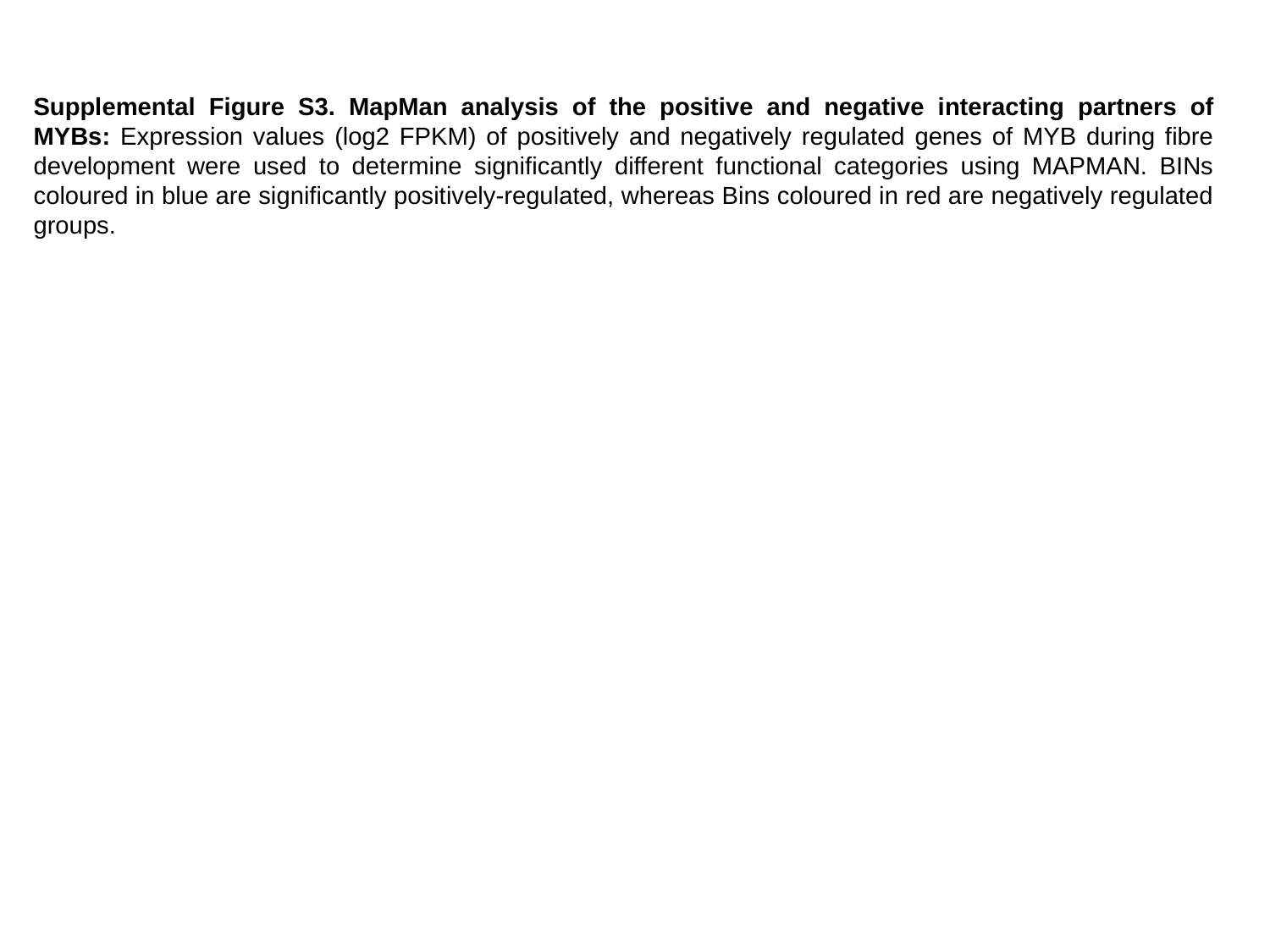

Supplemental Figure S3. MapMan analysis of the positive and negative interacting partners of MYBs: Expression values (log2 FPKM) of positively and negatively regulated genes of MYB during fibre development were used to determine significantly different functional categories using MAPMAN. BINs coloured in blue are significantly positively-regulated, whereas Bins coloured in red are negatively regulated groups.

## Slide 9
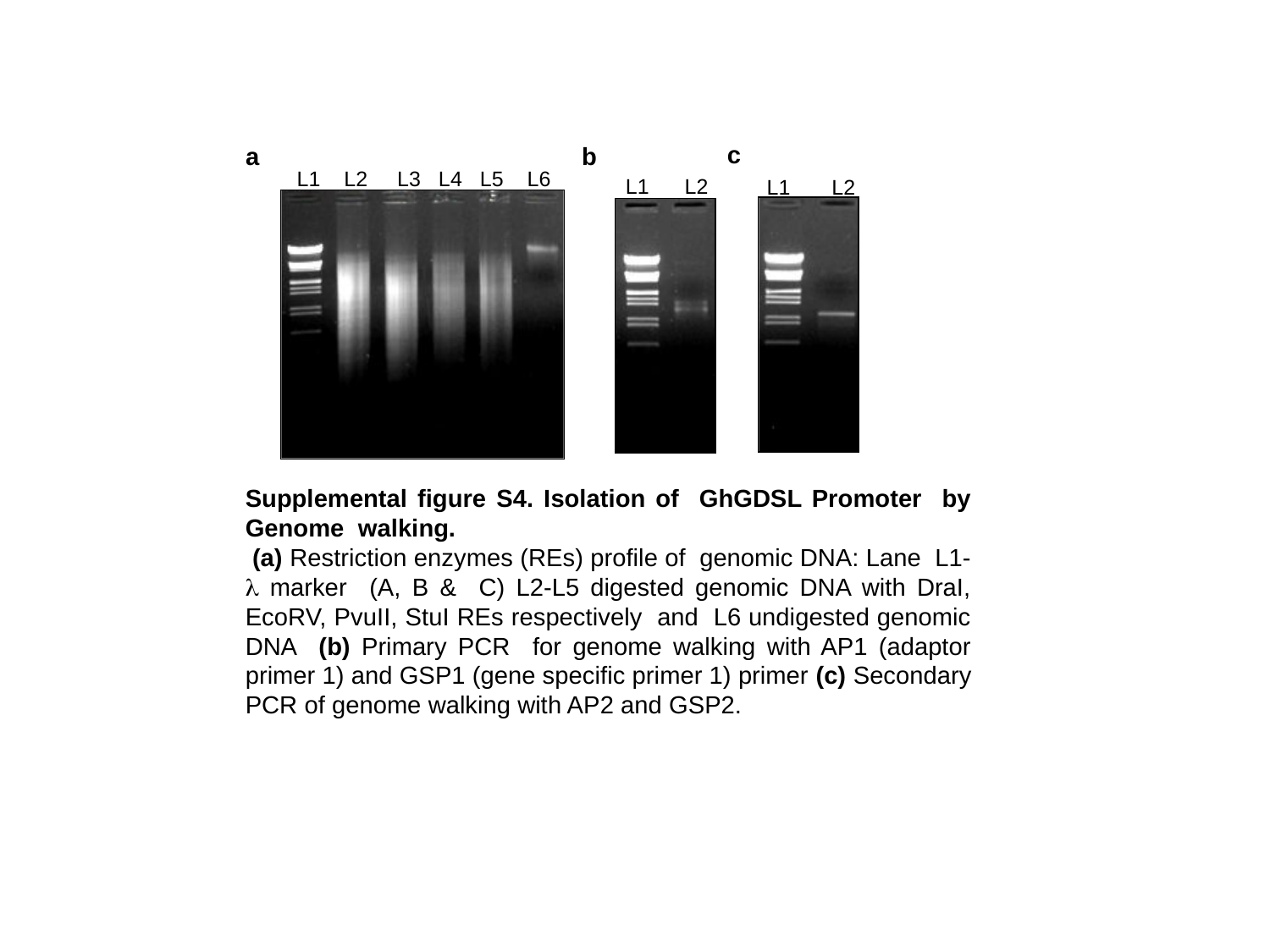

c
 L1 L2
b
L1 L2
a
L1 L2 L3 L4 L5 L6
Supplemental figure S4. Isolation of GhGDSL Promoter by Genome walking.
 (a) Restriction enzymes (REs) profile of genomic DNA: Lane L1- marker (A, B & C) L2-L5 digested genomic DNA with DraI, EcoRV, PvuII, StuI REs respectively and L6 undigested genomic DNA (b) Primary PCR for genome walking with AP1 (adaptor primer 1) and GSP1 (gene specific primer 1) primer (c) Secondary PCR of genome walking with AP2 and GSP2.

## Slide 10
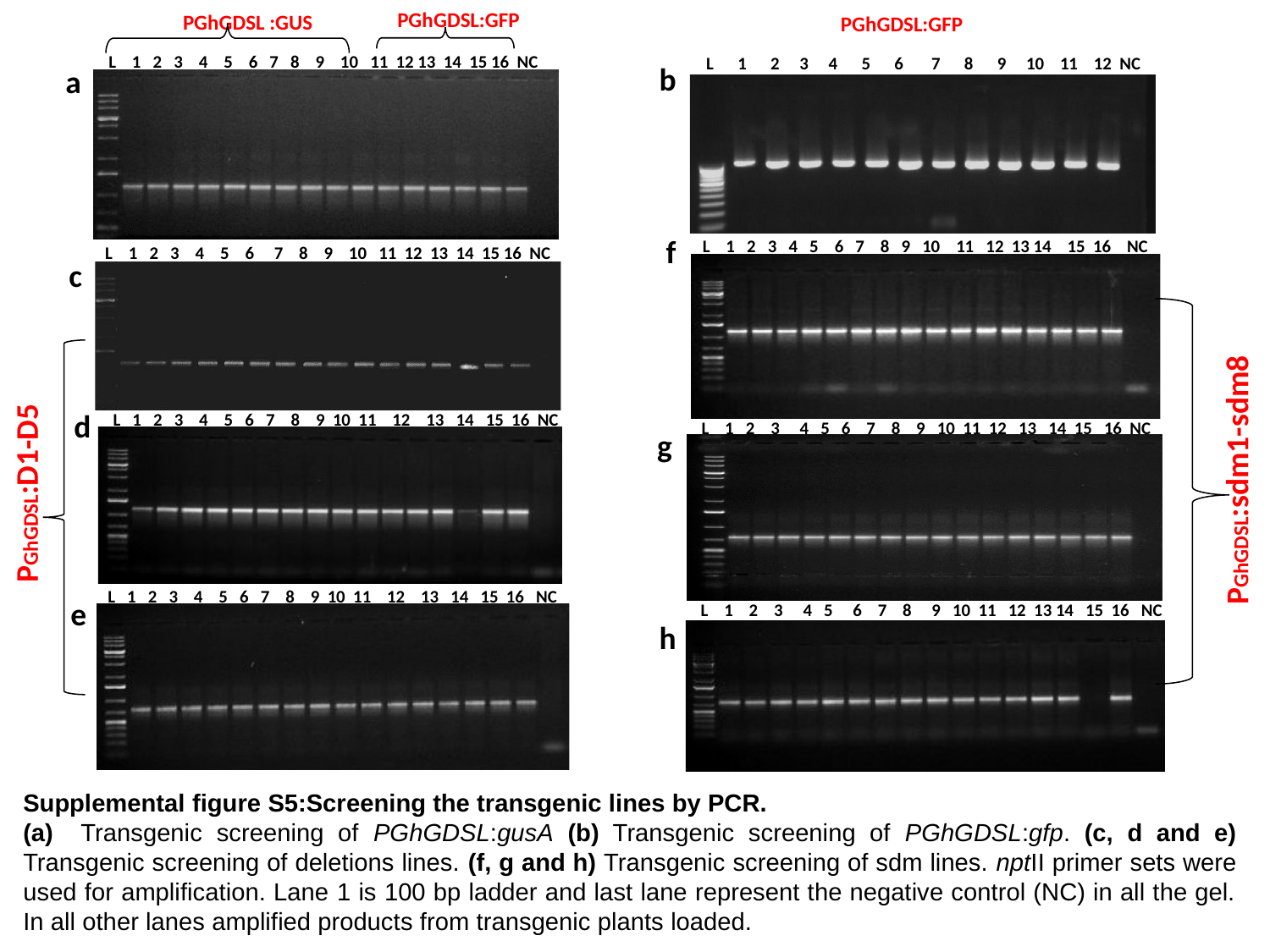

PGhGDSL:GFP
PGhGDSL :GUS
PGhGDSL:GFP
 L 1 2 3 4 5 6 7 8 9 10 11 12 13 14 15 16 NC
b
a
f
L 1 2 3 4 5 6 7 8 9 10 11 12 13 14 15 16 NC
 L 1 2 3 4 5 6 7 8 9 10 11 12 13 14 15 16 NC
c
d
L 1 2 3 4 5 6 7 8 9 10 11 12 13 14 15 16 NC
L 1 2 3 4 5 6 7 8 9 10 11 12 13 14 15 16 NC
g
PGhGDSL:sdm1-sdm8
PGhGDSL:D1-D5
L 1 2 3 4 5 6 7 8 9 10 11 12 13 14 15 16 NC
e
L 1 2 3 4 5 6 7 8 9 10 11 12 13 14 15 16 NC
h
 L 1 2 3 4 5 6 7 8 9 10 11 12 NC
Supplemental figure S5:Screening the transgenic lines by PCR.
(a) Transgenic screening of PGhGDSL:gusA (b) Transgenic screening of PGhGDSL:gfp. (c, d and e) Transgenic screening of deletions lines. (f, g and h) Transgenic screening of sdm lines. nptII primer sets were used for amplification. Lane 1 is 100 bp ladder and last lane represent the negative control (NC) in all the gel. In all other lanes amplified products from transgenic plants loaded.

## Slide 11
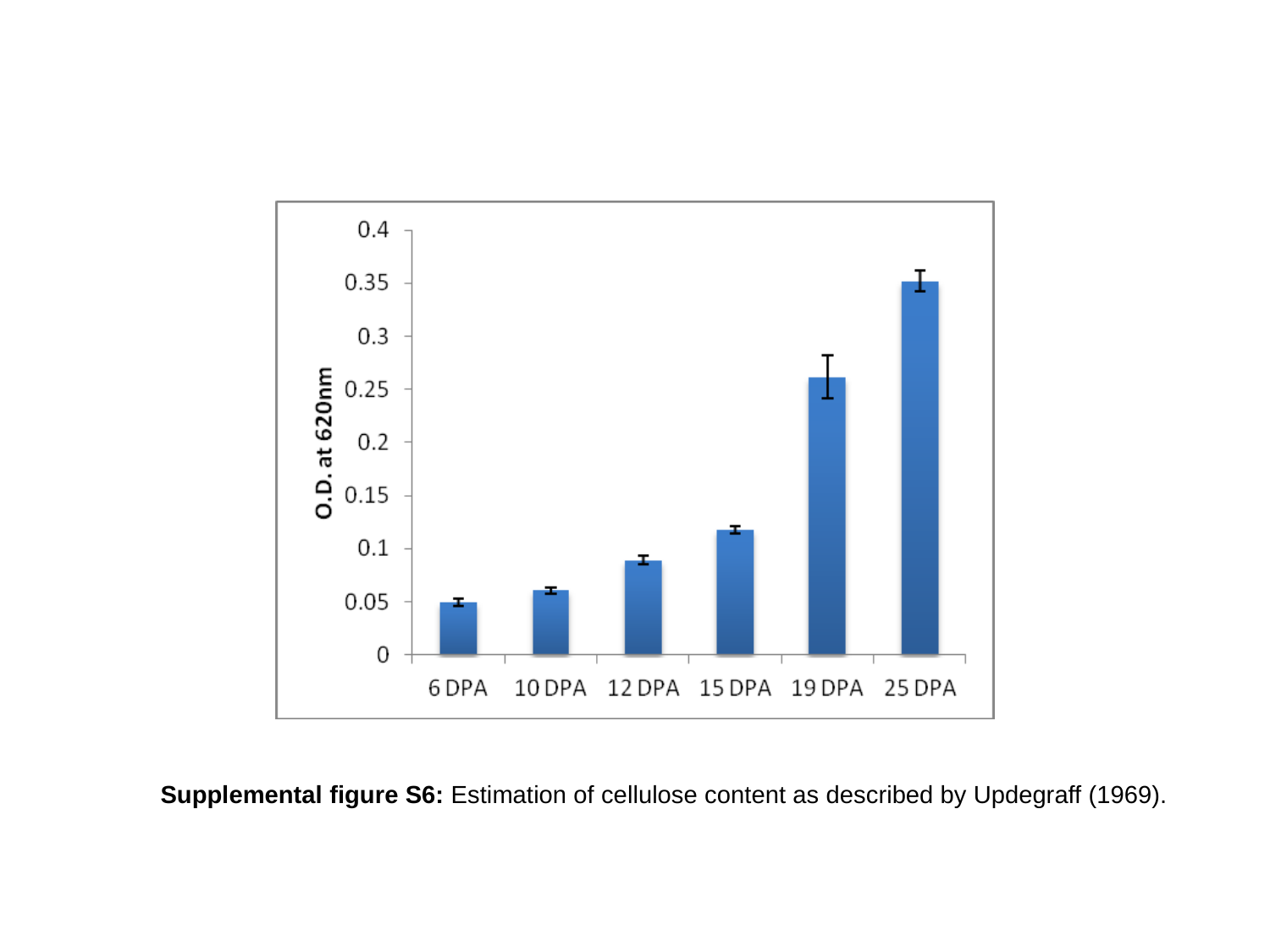

Supplemental figure S6: Estimation of cellulose content as described by Updegraff (1969).

## Slide 12
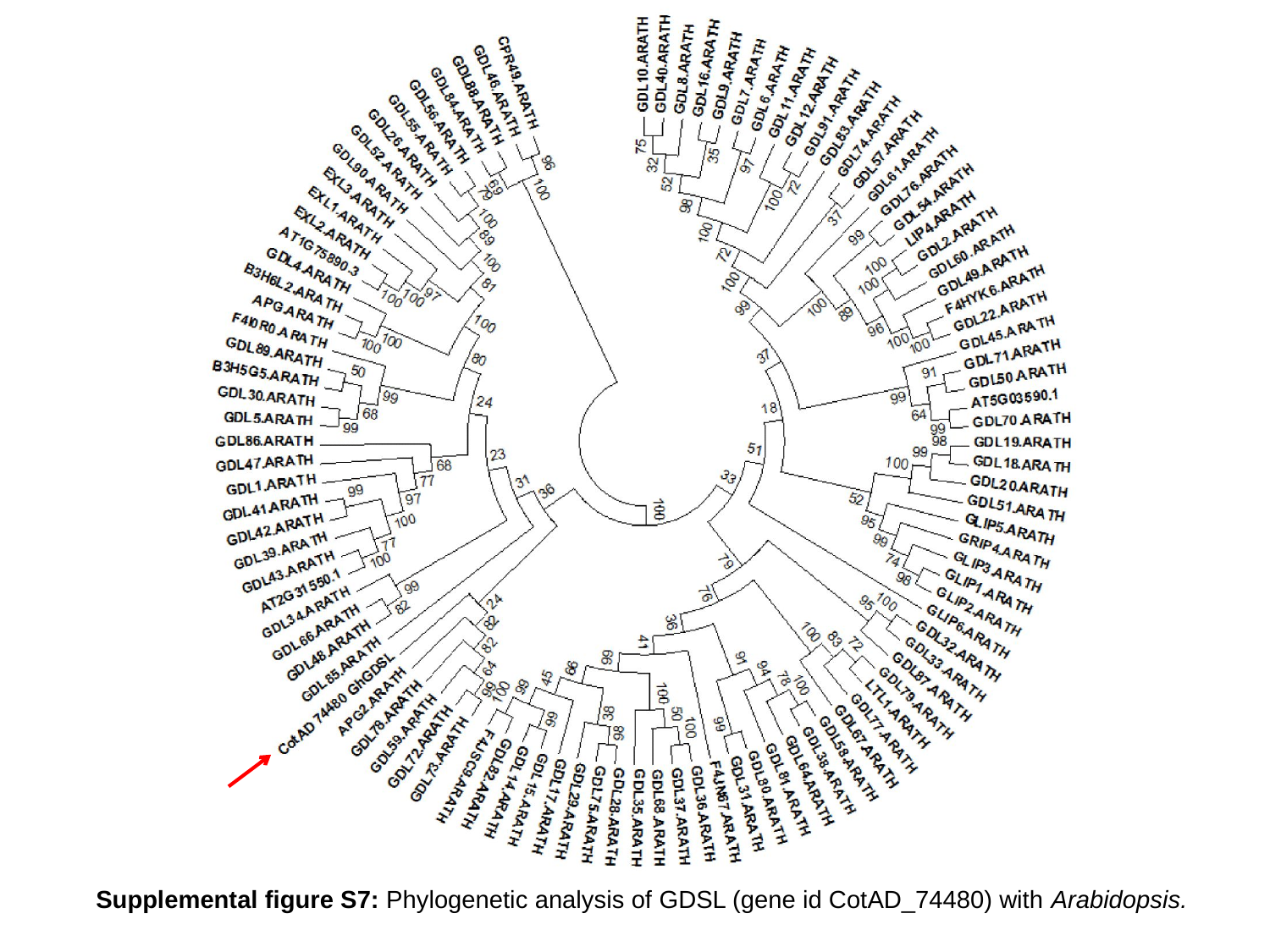

Supplemental figure S7: Phylogenetic analysis of GDSL (gene id CotAD_74480) with Arabidopsis.

## Slide 13
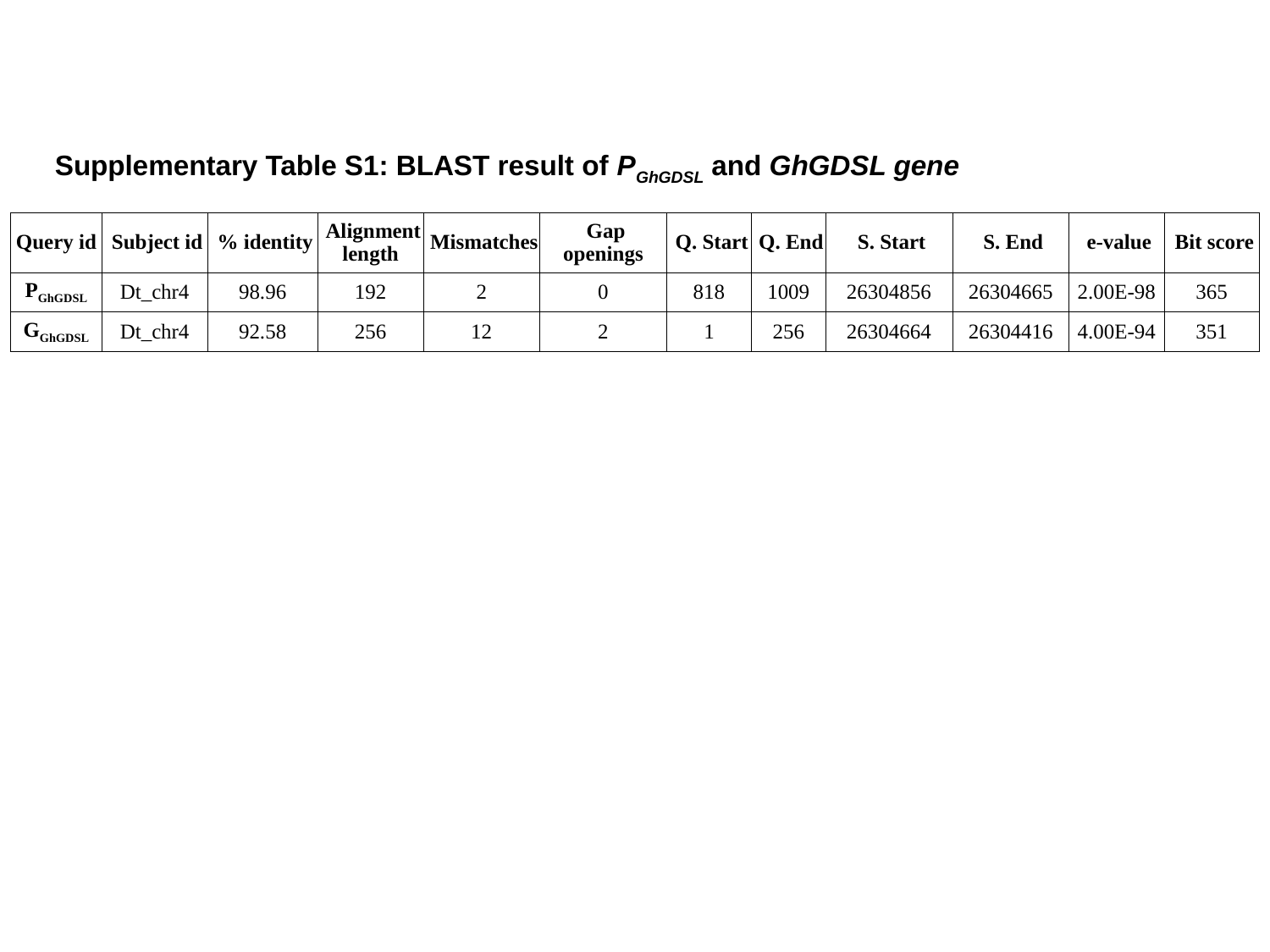

Supplementary Table S1: BLAST result of PGhGDSL and GhGDSL gene
| Query id | Subject id | % identity | Alignment length | Mismatches | Gap openings | Q. Start | Q. End | S. Start | S. End | e-value | Bit score |
| --- | --- | --- | --- | --- | --- | --- | --- | --- | --- | --- | --- |
| PGhGDSL | Dt\_chr4 | 98.96 | 192 | 2 | 0 | 818 | 1009 | 26304856 | 26304665 | 2.00E-98 | 365 |
| GGhGDSL | Dt\_chr4 | 92.58 | 256 | 12 | 2 | 1 | 256 | 26304664 | 26304416 | 4.00E-94 | 351 |

## Slide 14
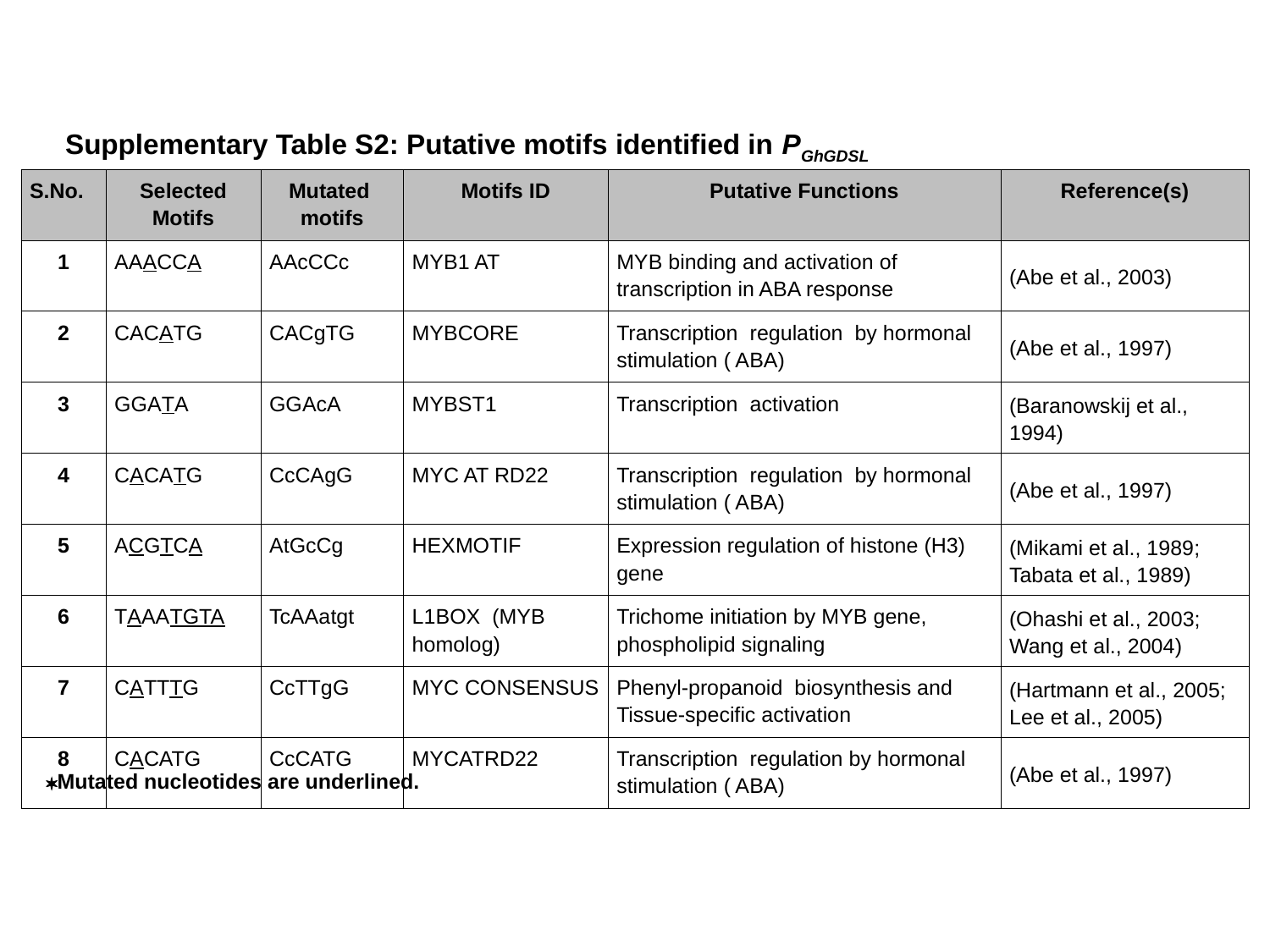

Supplementary Table S2: Putative motifs identified in PGhGDSL
| S.No. | Selected Motifs | Mutated motifs | Motifs ID | Putative Functions | Reference(s) |
| --- | --- | --- | --- | --- | --- |
| 1 | AAACCA | AAcCCc | MYB1 AT | MYB binding and activation of transcription in ABA response | (Abe et al., 2003) |
| 2 | CACATG | CACgTG | MYBCORE | Transcription regulation by hormonal stimulation ( ABA) | (Abe et al., 1997) |
| 3 | GGATA | GGAcA | MYBST1 | Transcription activation | (Baranowskij et al., 1994) |
| 4 | CACATG | CcCAgG | MYC AT RD22 | Transcription regulation by hormonal stimulation ( ABA) | (Abe et al., 1997) |
| 5 | ACGTCA | AtGcCg | HEXMOTIF | Expression regulation of histone (H3) gene | (Mikami et al., 1989; Tabata et al., 1989) |
| 6 | TAAATGTA | TcAAatgt | L1BOX (MYB homolog) | Trichome initiation by MYB gene, phospholipid signaling | (Ohashi et al., 2003; Wang et al., 2004) |
| 7 | CATTTG | CcTTgG | MYC CONSENSUS | Phenyl-propanoid biosynthesis and Tissue-specific activation | (Hartmann et al., 2005; Lee et al., 2005) |
| 8 | CACATG | CcCATG | MYCATRD22 | Transcription regulation by hormonal stimulation ( ABA) | (Abe et al., 1997) |
Mutated nucleotides are underlined.

## Slide 15
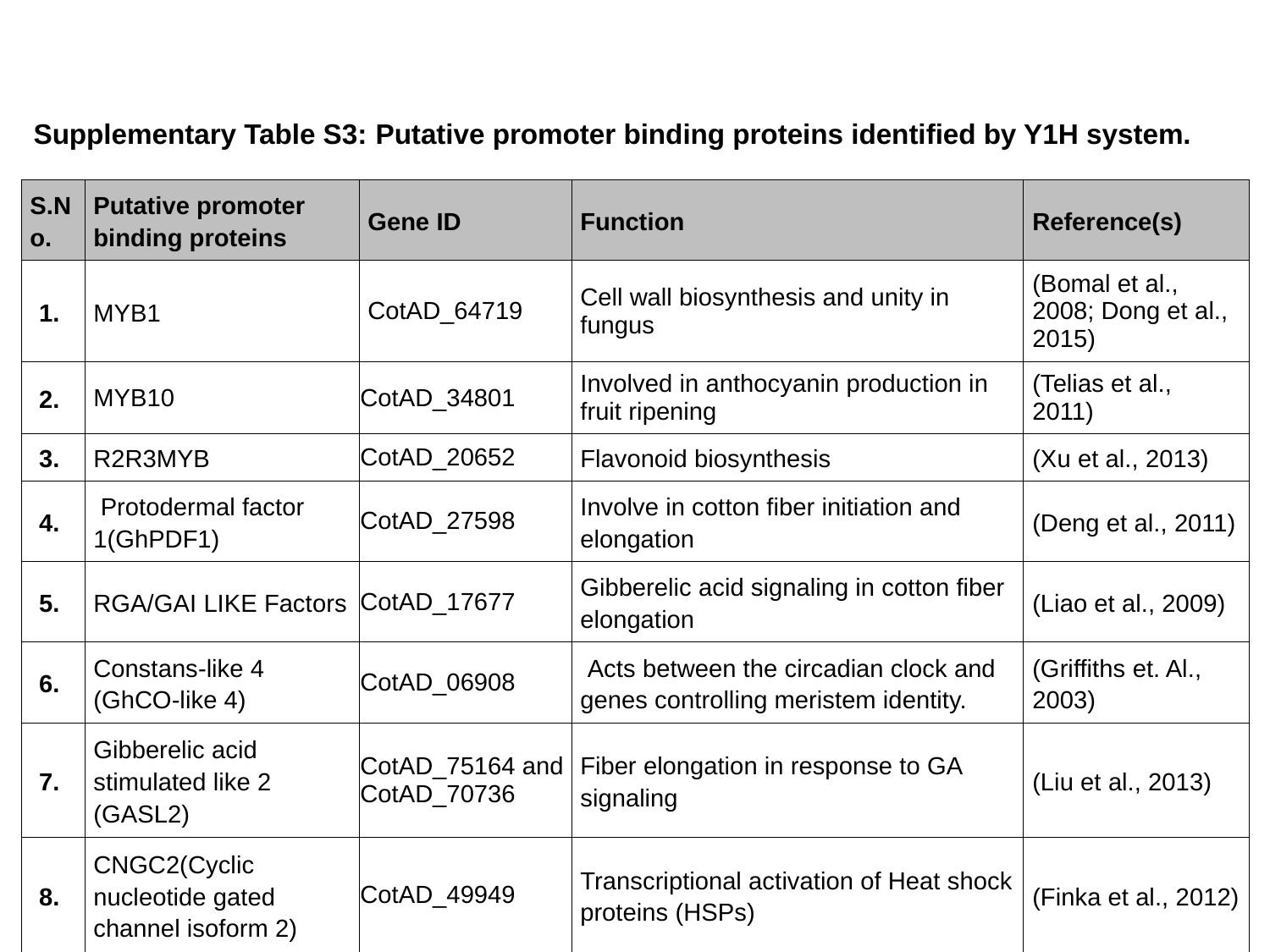

Supplementary Table S3: Putative promoter binding proteins identified by Y1H system.
| S.No. | Putative promoter binding proteins | Gene ID | Function | Reference(s) |
| --- | --- | --- | --- | --- |
| 1. | MYB1 | CotAD\_64719 | Cell wall biosynthesis and unity in fungus | (Bomal et al., 2008; Dong et al., 2015) |
| 2. | MYB10 | CotAD\_34801 | Involved in anthocyanin production in fruit ripening | (Telias et al., 2011) |
| 3. | R2R3MYB | CotAD\_20652 | Flavonoid biosynthesis | (Xu et al., 2013) |
| 4. | Protodermal factor 1(GhPDF1) | CotAD\_27598 | Involve in cotton fiber initiation and elongation | (Deng et al., 2011) |
| 5. | RGA/GAI LIKE Factors | CotAD\_17677 | Gibberelic acid signaling in cotton fiber elongation | (Liao et al., 2009) |
| 6. | Constans-like 4 (GhCO-like 4) | CotAD\_06908 | Acts between the circadian clock and genes controlling meristem identity. | (Griffiths et. Al., 2003) |
| 7. | Gibberelic acid stimulated like 2 (GASL2) | CotAD\_75164 and CotAD\_70736 | Fiber elongation in response to GA signaling | (Liu et al., 2013) |
| 8. | CNGC2(Cyclic nucleotide gated channel isoform 2) | CotAD\_49949 | Transcriptional activation of Heat shock proteins (HSPs) | (Finka et al., 2012) |
| 9. | FDH (Fiddle head like) | CotAD\_06815 | Trichome differentiation in Arabidopsis | (Yephremov et al., 1999) |

## Slide 16
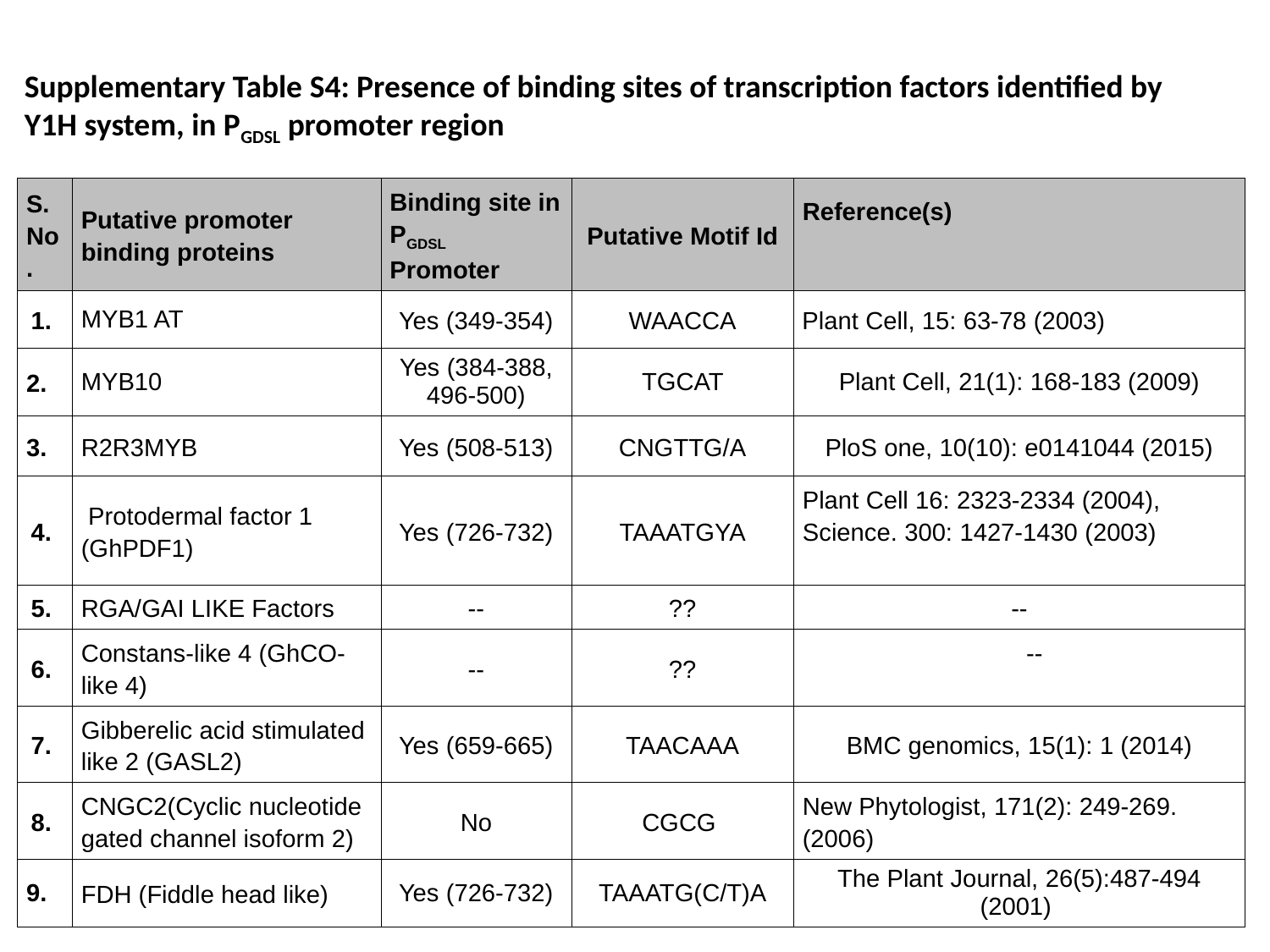

Supplementary Table S4: Presence of binding sites of transcription factors identified by Y1H system, in PGDSL promoter region
| S.No. | Putative promoter binding proteins | Binding site in PGDSL Promoter | Putative Motif Id | Reference(s) |
| --- | --- | --- | --- | --- |
| 1. | MYB1 AT | Yes (349-354) | WAACCA | Plant Cell, 15: 63-78 (2003) |
| 2. | MYB10 | Yes (384-388, 496-500) | TGCAT | Plant Cell, 21(1): 168-183 (2009) |
| 3. | R2R3MYB | Yes (508-513) | CNGTTG/A | PloS one, 10(10): e0141044 (2015) |
| 4. | Protodermal factor 1 (GhPDF1) | Yes (726-732) | TAAATGYA | Plant Cell 16: 2323-2334 (2004), Science. 300: 1427-1430 (2003) |
| 5. | RGA/GAI LIKE Factors | -- | ?? | -- |
| 6. | Constans-like 4 (GhCO-like 4) | -- | ?? | -- |
| 7. | Gibberelic acid stimulated like 2 (GASL2) | Yes (659-665) | TAACAAA | BMC genomics, 15(1): 1 (2014) |
| 8. | CNGC2(Cyclic nucleotide gated channel isoform 2) | No | CGCG | New Phytologist, 171(2): 249-269.(2006) |
| 9. | FDH (Fiddle head like) | Yes (726-732) | TAAATG(C/T)A | The Plant Journal, 26(5):487-494 (2001) |

## Slide 17
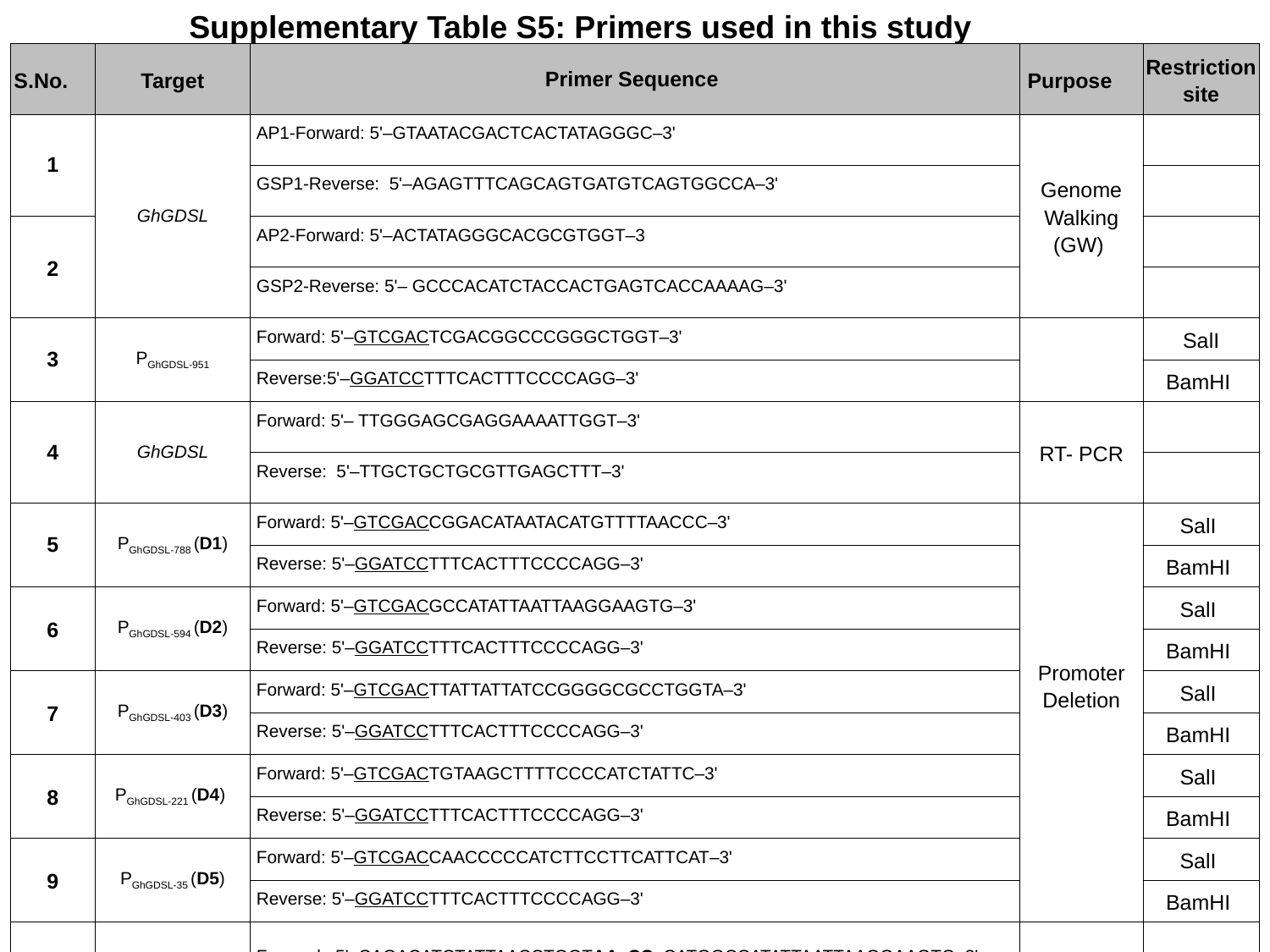

Supplementary Table S5: Primers used in this study
| S.No. | Target | Primer Sequence | Purpose | Restriction site |
| --- | --- | --- | --- | --- |
| 1 | GhGDSL | AP1-Forward: 5'–GTAATACGACTCACTATAGGGC–3' | Genome Walking (GW) | |
| | | GSP1-Reverse: 5'–AGAGTTTCAGCAGTGATGTCAGTGGCCA–3' | | |
| 2 | | AP2-Forward: 5'–ACTATAGGGCACGCGTGGT–3 | | |
| | | GSP2-Reverse: 5'– GCCCACATCTACCACTGAGTCACCAAAAG–3' | | |
| 3 | PGhGDSL-951 | Forward: 5'–GTCGACTCGACGGCCCGGGCTGGT–3' | | SalI |
| | | Reverse:5'–GGATCCTTTCACTTTCCCCAGG–3' | | BamHI |
| 4 | GhGDSL | Forward: 5'– TTGGGAGCGAGGAAAATTGGT–3' | RT- PCR | |
| | | Reverse: 5'–TTGCTGCTGCGTTGAGCTTT–3' | | |
| 5 | PGhGDSL-788 (D1) | Forward: 5'–GTCGACCGGACATAATACATGTTTTAACCC–3' | Promoter Deletion | SalI |
| | | Reverse: 5'–GGATCCTTTCACTTTCCCCAGG–3' | | BamHI |
| 6 | PGhGDSL-594 (D2) | Forward: 5'–GTCGACGCCATATTAATTAAGGAAGTG–3' | | SalI |
| | | Reverse: 5'–GGATCCTTTCACTTTCCCCAGG–3' | | BamHI |
| 7 | PGhGDSL-403 (D3) | Forward: 5'–GTCGACTTATTATTATCCGGGGCGCCTGGTA–3' | | SalI |
| | | Reverse: 5'–GGATCCTTTCACTTTCCCCAGG–3' | | BamHI |
| 8 | PGhGDSL-221 (D4) | Forward: 5'–GTCGACTGTAAGCTTTTCCCCATCTATTC–3' | | SalI |
| | | Reverse: 5'–GGATCCTTTCACTTTCCCCAGG–3' | | BamHI |
| 9 | PGhGDSL-35 (D5) | Forward: 5'–GTCGACCAACCCCCATCTTCCTTCATTCAT–3' | | SalI |
| | | Reverse: 5'–GGATCCTTTCACTTTCCCCAGG–3' | | BamHI |
| 10 | MYB1 AT (sdm1) | Forward: 5'–CAGACATCTATTAACCTGGTAAcCCcCATGGCCATATTAATTAAGGAAGTG–3' | Site Directed Mutagenesis (SDM) | |
| | | Reverse: 5'–CACTTCCTTAATTAATATGGCCATGgGGgTTACCAGGTTAATAGATGTCTG–3' | | |
| 11 | MYBCORE (sdm2) | Forward: 5'–CAGACATCTATTAACCTGGTAAACCACgTGGCCATATTAATTAAGGAAGTG–3' | | |
| | | Reverse: 5'–CACTTCCTTAATTAATATGGCCAcGTGGTTTACCAGGTTAATAGATGTCTG–3' | | |
| 12 | MYBST1 (sdm2) | Forward: 5'–GCATTTTGTTGGAcACTTAACCTTCTTTTTTGGGGATGAATGTG–3' | | |
| | | Reverse: 5'–CACATTCATCCCCAAAAAAGAAGGTTAAGTgTCCAACAAAATGC–3' | | |
| | | | | |
| 13 | MYC AT RD22 (sdm3) | Forward: 5'–AATAACTCCTGCATGCcCAgGCAGTTGCTCTT–3' | | |
| | | Reverse: 5'–AAGAGCAACTGCcTGgGCATGCAGGAGTTATT–3' | | |
| 14 | HEXMOTIF (sdm4) | Forward: 5'–TTGCTCTTTCGTTCCACACCATGAAtGcCgCTGCTTT–3' | | |
| | | Reverse: 5'–AAAGCAGcGgCaTTCATGGTGTGGAACGAAAGAGCAA–3' | | |

## Slide 18
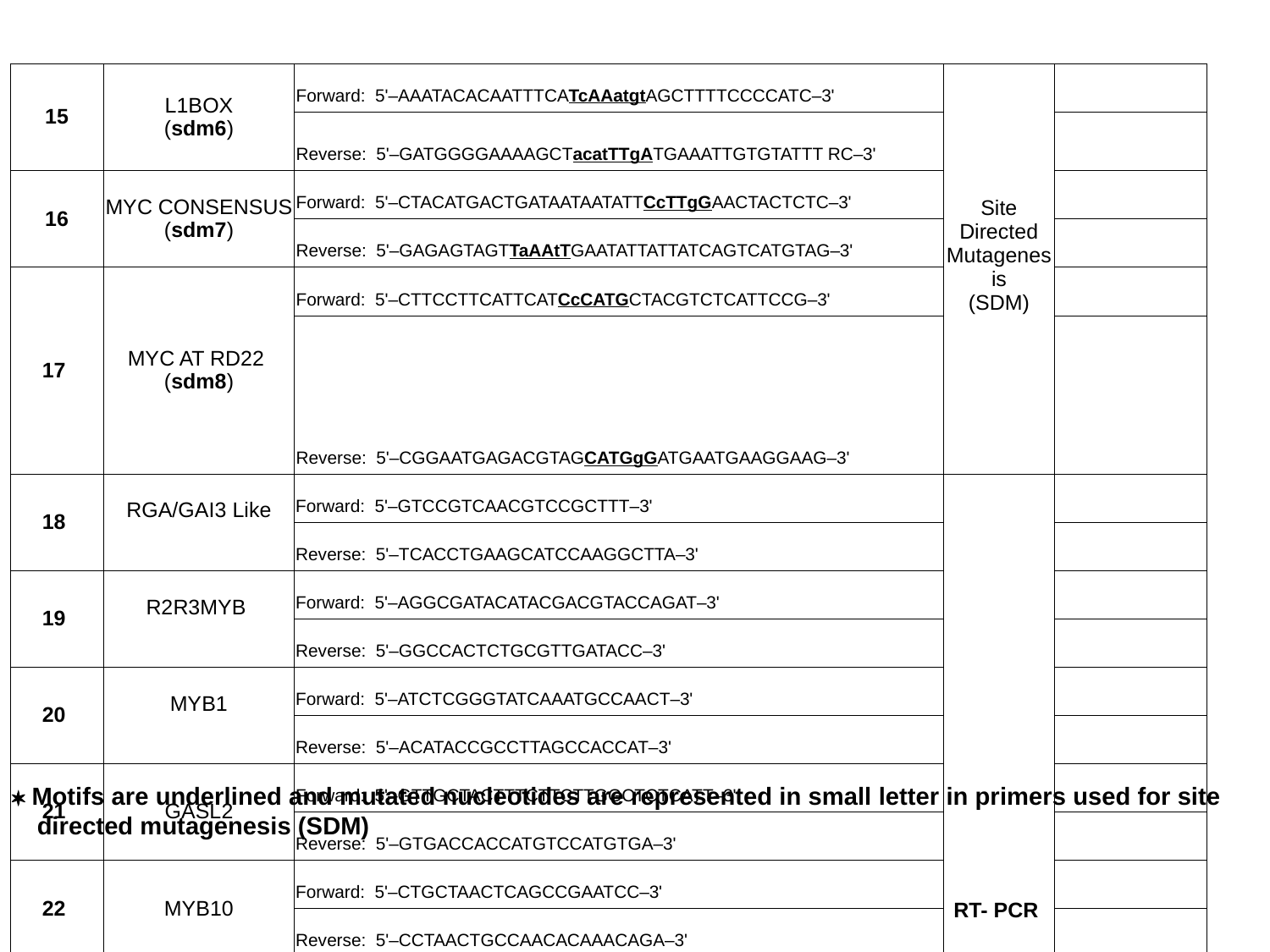

| 15 | L1BOX (sdm6) | Forward: 5'–AAATACACAATTTCATcAAatgtAGCTTTTCCCCATC–3' | Site Directed Mutagenesis (SDM) | |
| --- | --- | --- | --- | --- |
| | | Reverse: 5'–GATGGGGAAAAGCTacatTTgATGAAATTGTGTATTT RC–3' | | |
| 16 | MYC CONSENSUS (sdm7) | Forward: 5'–CTACATGACTGATAATAATATTCcTTgGAACTACTCTC–3' | | |
| | | Reverse: 5'–GAGAGTAGTTaAAtTGAATATTATTATCAGTCATGTAG–3' | | |
| 17 | MYC AT RD22 (sdm8) | Forward: 5'–CTTCCTTCATTCATCcCATGCTACGTCTCATTCCG–3' | | |
| | | Reverse: 5'–CGGAATGAGACGTAGCATGgGATGAATGAAGGAAG–3' | | |
| 18 | RGA/GAI3 Like | Forward: 5'–GTCCGTCAACGTCCGCTTT–3' | RT- PCR | |
| | | Reverse: 5'–TCACCTGAAGCATCCAAGGCTTA–3' | | |
| 19 | R2R3MYB | Forward: 5'–AGGCGATACATACGACGTACCAGAT–3' | | |
| | | Reverse: 5'–GGCCACTCTGCGTTGATACC–3' | | |
| 20 | MYB1 | Forward: 5'–ATCTCGGGTATCAAATGCCAACT–3' | | |
| | | Reverse: 5'–ACATACCGCCTTAGCCACCAT–3' | | |
| 21 | GASL2 | Forward: 5'–GTTGCTACTTTCTTCTTGGCTCTCATT–3' | | |
| | | Reverse: 5'–GTGACCACCATGTCCATGTGA–3' | | |
| 22 | MYB10 | Forward: 5'–CTGCTAACTCAGCCGAATCC–3' | | |
| | | Reverse: 5'–CCTAACTGCCAACACAAACAGA–3' | | |
| 23 | GhCO-Like 4 | Forward: 5'–GGGTCTTGGTCTGTGAAGTTTGC–3' | | |
| | | Reverse: 5'–GAGTGGATGTCTCGGTCACAAGT–3' | | |
| 24 | CNGC2 | Forward: 5'–CTTCTCAGTCCAACTTCTCCCTATCA–3' | | |
| | | Reverse: 5'–ATTATCAGATCTCTCTGGCATTGAGT–3' | | |
| 25 | GhPDF1 | Forward: 5'–TCCCATACGACGGTACCAGATT–3' | | |
| | | Reverse: 5'–TAATGGCCACTCTGCGTTGATAC–3' | | |
| 26 | FDH | Forward: 5'–CCTCCCCTTCTCCGAACAACT–3' | | |
| | | Reverse: 5'–GGTGATAGGGAGGTCTTGGATTTATC–3' | | |
 Motifs are underlined and mutated nucleotides are represented in small letter in primers used for site directed mutagenesis (SDM)

## Slide 19
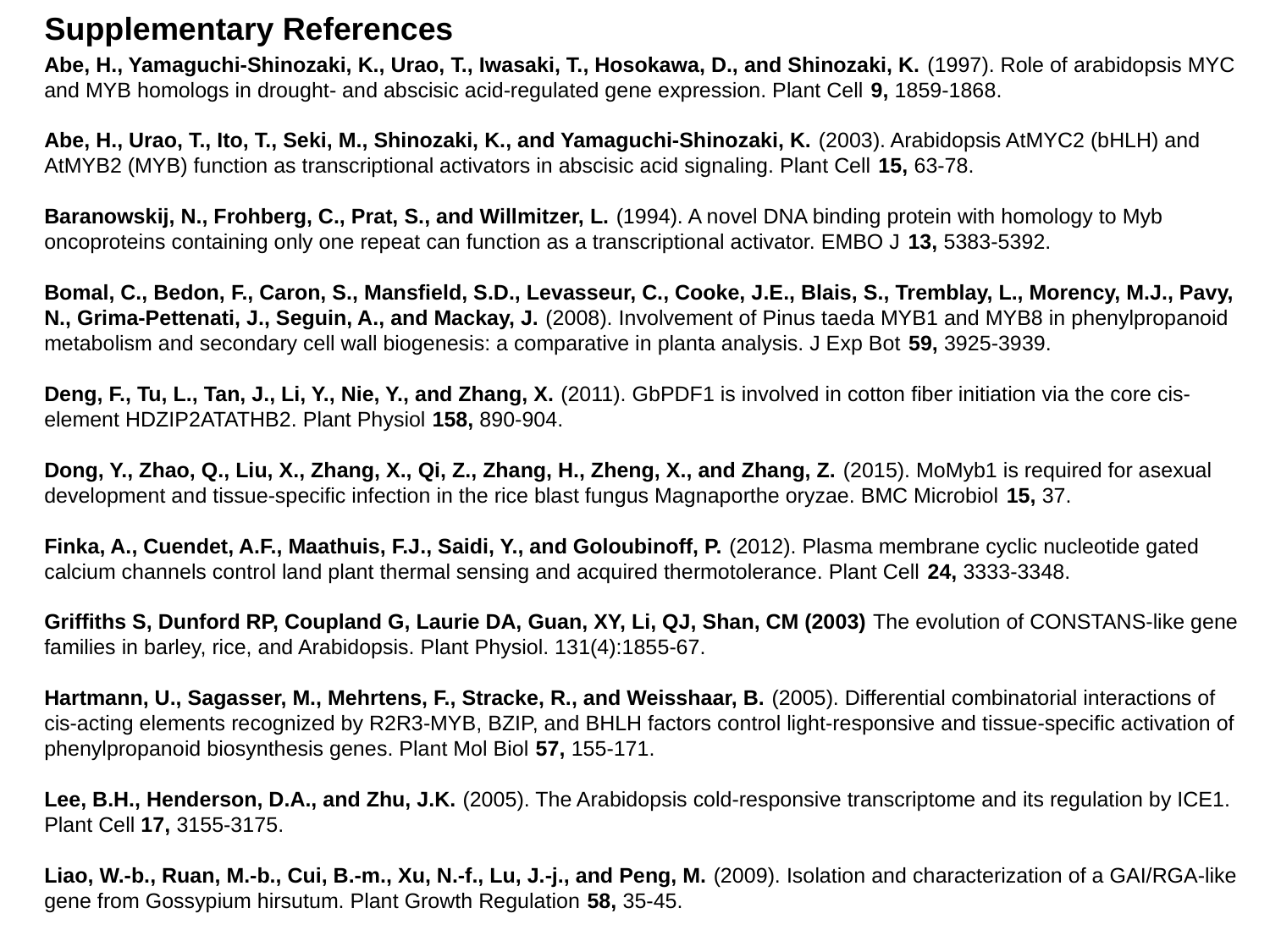

Supplementary References
Abe, H., Yamaguchi-Shinozaki, K., Urao, T., Iwasaki, T., Hosokawa, D., and Shinozaki, K. (1997). Role of arabidopsis MYC and MYB homologs in drought- and abscisic acid-regulated gene expression. Plant Cell 9, 1859-1868.
Abe, H., Urao, T., Ito, T., Seki, M., Shinozaki, K., and Yamaguchi-Shinozaki, K. (2003). Arabidopsis AtMYC2 (bHLH) and AtMYB2 (MYB) function as transcriptional activators in abscisic acid signaling. Plant Cell 15, 63-78.
Baranowskij, N., Frohberg, C., Prat, S., and Willmitzer, L. (1994). A novel DNA binding protein with homology to Myb oncoproteins containing only one repeat can function as a transcriptional activator. EMBO J 13, 5383-5392.
Bomal, C., Bedon, F., Caron, S., Mansfield, S.D., Levasseur, C., Cooke, J.E., Blais, S., Tremblay, L., Morency, M.J., Pavy, N., Grima-Pettenati, J., Seguin, A., and Mackay, J. (2008). Involvement of Pinus taeda MYB1 and MYB8 in phenylpropanoid metabolism and secondary cell wall biogenesis: a comparative in planta analysis. J Exp Bot 59, 3925-3939.
Deng, F., Tu, L., Tan, J., Li, Y., Nie, Y., and Zhang, X. (2011). GbPDF1 is involved in cotton fiber initiation via the core cis-element HDZIP2ATATHB2. Plant Physiol 158, 890-904.
Dong, Y., Zhao, Q., Liu, X., Zhang, X., Qi, Z., Zhang, H., Zheng, X., and Zhang, Z. (2015). MoMyb1 is required for asexual development and tissue-specific infection in the rice blast fungus Magnaporthe oryzae. BMC Microbiol 15, 37.
Finka, A., Cuendet, A.F., Maathuis, F.J., Saidi, Y., and Goloubinoff, P. (2012). Plasma membrane cyclic nucleotide gated calcium channels control land plant thermal sensing and acquired thermotolerance. Plant Cell 24, 3333-3348.
Griffiths S, Dunford RP, Coupland G, Laurie DA, Guan, XY, Li, QJ, Shan, CM (2003) The evolution of CONSTANS-like gene families in barley, rice, and Arabidopsis. Plant Physiol. 131(4):1855-67.
Hartmann, U., Sagasser, M., Mehrtens, F., Stracke, R., and Weisshaar, B. (2005). Differential combinatorial interactions of cis-acting elements recognized by R2R3-MYB, BZIP, and BHLH factors control light-responsive and tissue-specific activation of phenylpropanoid biosynthesis genes. Plant Mol Biol 57, 155-171.
Lee, B.H., Henderson, D.A., and Zhu, J.K. (2005). The Arabidopsis cold-responsive transcriptome and its regulation by ICE1. Plant Cell 17, 3155-3175.
Liao, W.-b., Ruan, M.-b., Cui, B.-m., Xu, N.-f., Lu, J.-j., and Peng, M. (2009). Isolation and characterization of a GAI/RGA-like gene from Gossypium hirsutum. Plant Growth Regulation 58, 35-45.

## Slide 20
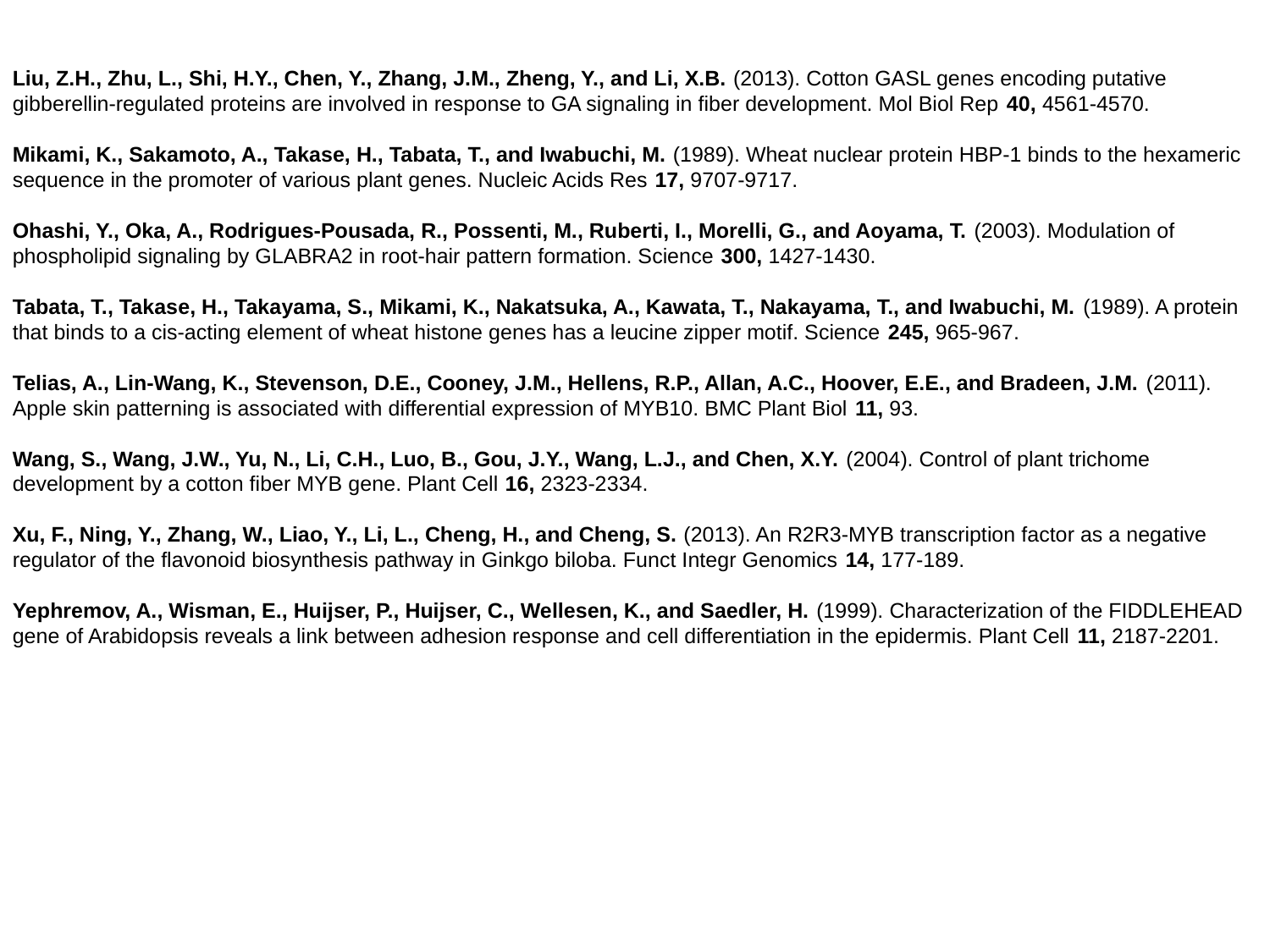

Liu, Z.H., Zhu, L., Shi, H.Y., Chen, Y., Zhang, J.M., Zheng, Y., and Li, X.B. (2013). Cotton GASL genes encoding putative gibberellin-regulated proteins are involved in response to GA signaling in fiber development. Mol Biol Rep 40, 4561-4570.
Mikami, K., Sakamoto, A., Takase, H., Tabata, T., and Iwabuchi, M. (1989). Wheat nuclear protein HBP-1 binds to the hexameric sequence in the promoter of various plant genes. Nucleic Acids Res 17, 9707-9717.
Ohashi, Y., Oka, A., Rodrigues-Pousada, R., Possenti, M., Ruberti, I., Morelli, G., and Aoyama, T. (2003). Modulation of phospholipid signaling by GLABRA2 in root-hair pattern formation. Science 300, 1427-1430.
Tabata, T., Takase, H., Takayama, S., Mikami, K., Nakatsuka, A., Kawata, T., Nakayama, T., and Iwabuchi, M. (1989). A protein that binds to a cis-acting element of wheat histone genes has a leucine zipper motif. Science 245, 965-967.
Telias, A., Lin-Wang, K., Stevenson, D.E., Cooney, J.M., Hellens, R.P., Allan, A.C., Hoover, E.E., and Bradeen, J.M. (2011). Apple skin patterning is associated with differential expression of MYB10. BMC Plant Biol 11, 93.
Wang, S., Wang, J.W., Yu, N., Li, C.H., Luo, B., Gou, J.Y., Wang, L.J., and Chen, X.Y. (2004). Control of plant trichome development by a cotton fiber MYB gene. Plant Cell 16, 2323-2334.
Xu, F., Ning, Y., Zhang, W., Liao, Y., Li, L., Cheng, H., and Cheng, S. (2013). An R2R3-MYB transcription factor as a negative regulator of the flavonoid biosynthesis pathway in Ginkgo biloba. Funct Integr Genomics 14, 177-189.
Yephremov, A., Wisman, E., Huijser, P., Huijser, C., Wellesen, K., and Saedler, H. (1999). Characterization of the FIDDLEHEAD gene of Arabidopsis reveals a link between adhesion response and cell differentiation in the epidermis. Plant Cell 11, 2187-2201.
