# Supplementary material for: GhMYB1 regulates SCW stage‐specific expression of the GhGDSL promoter in the fibres of Gossypium hirsutum L
Source: Plant Biotechnol J. 2017 Mar 23;15(9):1163–74. doi: 10.1111/pbi.12706 (PMC5552479; doi:10.1111/pbi.12706)
Supplement: Supplementary file 3 — Appendix S2 Supplementary materials and methods. [file PBI-15-1163-s001.doc]

**GhMYB1 regulate SCW stage-specific expression of the *GhGDSL* promoter in the fibers of *Gossypium hirsutum L.***

Vrijesh Kumar Yadav, Vikash Kumar Yadav, Poonam Pant, Surendra Pratap Singh, Rashmi Maurya, Anshulika Sable, Samir V. Sawant^*^

**Supplemental Materials and Methods**

**Microarray and Real-time PCR (RT-PCR) analysis**

We previously reported microarray profiling of five contrasting genotypes (JKC725, JKC777, JKC703, JKC737, and JKC783) of *G. hirsutum* on an Affymetrix cotton chip. This profiling included the fiber developmental stages such as initiation (0DPA), elongation (6, 9, and 12 DPA) and SCW biosynthesis (19 and 25 DPA) (Nigam et al., 2013). These five contrasting genotypes included two superior (JKC725 and JKC777) and three inferior (JKC703, JKC737, and JKC783) genotypes based on their fiber quality parameters. The *GhGDSL* gene was sorted from microarray data (probe ID Ghi.8746.2. A1_x_at). The average normalized intensity values of the five contrasting genotypes (JKC725, JKC777, JKC703, JKC737, and JKC783) from microarray data of *GhGDSL* were plotted.

Total RNA from the different fiber developmental stages, such as initiation (0 DPA), elongation (6, 9, and 12 DPA) and SCW biosynthesis (19 and 25 DPA) of *G. hirsutum*, was extracted using Qiagen RNAeasy Plant Mini Kit (Qiagen, Germantown, MD, USA). The quantity and quality of RNA was assessed by using Nano-Drop (Thermo Scientific Wilmington, DE, USA) and agarose gel electrophoresis. The total RNA was subjected to DNase I (Invitrogen, Carlsbad, CA, USA) treatment to ensure the removal of any residual DNA, which may interfere the downstream expression analysis. Further by using SuperScript ® II reverse transcriptase kit (Invitrogen, Carlsbad, CA, USA) cDNA was synthesized. The cDNAs was prepared from 2 µg of RNA and used as template in the qRT-PCR analysis. The gene-specific primers were designed by using Primer Express 3 software (Applied Biosystems, Foster City, CA, USA) and qRT-PCR was performed using a 7500 fast real-time PCR system (Applied Biosystems, Foster City, CA, USA) with SYBR green universal PCR master mix (Invitrogen, Carlsbad, CA, USA). The cotton polyubiquitin gene (EU604080) was used as an internal control. The transcript (mRNA expression) levels were quantified by using the ΔΔCt quantitative method (Livak and Schmittgen, 2001). The primers used are listed in Table S4.

**Genome walking and sequence analysis**

The total genomic DNA was extracted from cotton plant leaf tissues using cetyltrimethylammonium bromide (CTAB) method as described elsewhere (Porebski and Lynen, 2014). In this study Genome Walker ^TM^ Universal (GWU) Kits (Clontech, USA, Cat. No. 638904) was utilized to amplify the upstream unknown sequence of *GhGDSL* gene from its ORF (open reading frame). Four blunt end restriction enzymes (*StuI, DraI, PvuII* and *EcoRV*) were used to generate four adaptor-ligated genome walking libraries and one positive control human genomic DNA library (provided in the kit). The primary PCR was set up using gene-specific primers GSP1 and adapter primer AP1; and the PCR products were checked on an agarose gel (Figure S4a). The pri­mary PCR mixture was then diluted (49:1 ratio) and used as a template for the secondary PCR. The secondary PCR was carried out using adapter primer AP2 and gene-specific primer GSP2; and the PCR products were checked on an agarose gel (Figure S4b). The expected band was extracted from the gel using the NucleoSpin gel extraction and PCR cleanup kit (Machery Nagel, Clontech, USA). The isolated upstream DNA fragment was cloned into the pBluescript SK plus vector (Stratagene). The pBluescript SK plus vector specific forward primers T3 (5-AATTAACCCTCACTAAAGGG–3) and reverse primer T7 (5-GTAATACGACTCACTATAGGGC–3) were used for sequencing by a 96-capillary automated sequencer (ABI–3730 DNA Analyzer, UK). The primers used for the genome walking are listed in Table S5.

**Histochemical GUS staining and GUS assay**

The transgenic cotton lines were analyzed by a histochemical assay as described by Jefferson et al. (1987). The transgenic bolls from 0 to 25 DPA were incubated in 5-bromo-4-chloro-3-indolylglucuronide (X-gluc) solution at 37°C for overnight. The stained cotton bolls were cleared with 70% and 100% ethanol and viewed under a Leica/Wild M3Z stereo microscope. The GUS activity was measured according to Jefferson et al. (1987). The transgenic cotton bolls from the different developmental stages (0DPA to 25DPA) and leaf tissues were crushed in liquid nitrogen and suspended in 1 mL of GUS extraction buffer (1 M sodium phosphate pH 7.0, 0.5 M EDTA, 1 M DTT, 10% Triton X–100, 20% sarcosyl) and centrifuged at 13000 rpm for 15 min at the ambient temperature. A 10-µL aliquot of the supernatants was taken and mixed with 130 µL assay buffer (1 mM MUG in extraction buffer) and incubated the reaction at 37°C for 20 min in dark followed by termination using a stop buffer (0.04 M Na_2_CO_3_ ). The GUS activity was measured on a spectrophotometer (Synergy™ H1 Multi-Mode Reader) with the excitation at 455 nm and emission at 365 nm. Protein concentrations were determined as described by Bradford (1976) using the Bradford reagent (Bio-Rad, Hercules, CA, USA). Three biological replicates were taken to measure the GUS activity from 7–10 independent transgenic lines (T1) of cotton.

**Cellulose estimation**

Cellulose content was estimated as described by Updegraff (1969). The cotton fibers were dissected using sterile forceps from the ovules at the different developmental stages. The fibers were collected in a pre-chilled mortar and were ground finely in liquid nitrogen using a pestle. The ground tissue was transferred to 50 mL falcon tubes and rinsed with 10 mL of MilliQ water by centrifugation at 3500 ×g for 10 min. The pellet was incubated for 30 min with 3 mL of CH_3_COOH + HNO_3_ reagent (80% CH_3_COOH and HNO_3_ in 10:1 ratio) at 100 °C in a water bath. After incubation, the samples were centrifuged at 3500 ×g for 5 min. The pellet was rinsed thoroughly with MilliQ water. Furthermore, the pellet was treated with 10 mL of concentrated H_2_SO_4_ along with 500 mg of pure cellulose sample for reference and incubated for 1 h at the ambient temperature. Each of the hydrolyzed samples (100 μL) was diluted ten times with MilliQ. Similarly, the reference sample of the hydrolyzed cellulose was diluted to make a stock solution of 10 mg/mL, which was further diluted by 2−6 folds to make a standard graph. A total of 100 μL of these samples was treated with 200 μL of anthrone reagent (0.2% anthrone in conc. H_2_SO_4_) in 96-well flat-bottom plates and incubated for 15 min at the ambient temperature. The absorbance was measured at 620 nm in an Infinite M200 plate reader (Tecan, Latvia). The cellulose content in cotton fiber was calculated on the basis of the absorbance of the sample and the standard graph was made by pure cellulose sample.

**Microtomy and light microscopy**

Cotton ovules from different developmental stages (initiation, elongation, and SCW) were stained by 0.1% (w/v) X-gluc (Clontech, Terra Bella, CA**,** USA) as described by Jefferson et al. (1987). The stained ovules were incubated for 24 h in a prechilled Farmer’s fixative (Ethanol: Glacial acetic acid, 3:1) with a replacement after 12 h during the incubation. The samples were dehydrated using different concentrations of dehydration solution (Distilled water: absolute ethanol: tertbutyl alcohol) in a series in the ratio of 50:40:10 followed by 30:50:20, 15:50:35, 0:45:55, and 0:25:75 (100%) each for 1 h and finally in the absolute TBA (0:0:100) for 2 h. The tubes were incubated at 60 °C for TBA evaporation followed by addition of melted paraffin wax. Paraffin blocks were prepared and 4–6 μm thick sections were cut using the microtome (Leica), followed by rinsing in 4% formalin and incubation in 100% xylene for overnight. Images were captured from 0 to 25 DPA using a Nikon microscope camera (DXM1200F, Japan).

**Fluorescence of GFP expressed in cotton fiber**

The 951 nucleotide long fragment (*P_GhGDSL–9_*_51_), upstream to the start codon of *GhGDSL* gene was ligated into the plant expression vector pBI121-*gfp*. In this construct, GFP was sub-cloned in pBI121 by replacing *gus* and CaMV35 promoter was replaced by *P_GhGDSL–9_*_51_. After confirming the ligation of *P_GhGDSL–9_*_51_ by restriction enzyme digestion, it was transformed into cotton by *Agrobacterium*-mediated transformation. Positive plants were confirmed by PCR using the forward primer 5'-ATGATTGAACAAGATGGATTGCACG-3' (Npt–2 forward primer) and the reverse primer 5'-TCAGAAGAACTCGTCAAGAAGGC-3' (Npt–2 reverse primer). Fluorescence by GFP in cotton fiber during the different developmental stages (0DPA to 25DPA), such as initiation, elongation, and SCW, was captured (excitation wavelength 488 nm and emission filter 505/75 nm) using confocal laser microscope (LSM510 META, Carl Zeiss, Heidelberg, Germany).

**Transformation of cotton**

The transformation method for cotton established by Kumar et al. (2013) was used to generate cotton transgenics for functional characterization of the genes and promoters. *P_GhGDSL_*, all the deletions construct pBI-*P_GhGDSL_*_-D_: *gus* (D1, D2, D3, D4, and D5), mutagenic construct pBI-*P_GhGDSL_*_-M_: *gus* (sdm1 to sdm8), GFP construct pBI-*P_GhGDSL_*:*gfp* and the control vector were introduced into *A. tumefaciens* by the freeze-thaw method (Hofgen and Willmitzer, 1988). A single colony of *A.* *tumefaciens* LBA 4404 having binary vector was subjected for the selection using streptomycin, (250 μg/mL), rifampicin (50 μg/mL), and kanamycin (100 μg/mL) in YEB medium. Coker–312 seeds were surface sterilized with 0.1% HgCl_2_ solution for 5 min. The sterilized seeds were grown under moist condition and the germinated seeds were used for embryo transformation (Kumar et al., 2013). The germinated cotton seedlings were wounded at the apex side with a scalpel and infected overnight with *Agrobacterium* suspension culture. The infected seedlings were transferred on co-cultivation medium (1/2 MS) plates for 48 h at 28 °C in dark. After two days, the seedlings were washed with cefotaxime (250 mg/L) for 5 min followed by four rinses with autoclaved MilliQ water. The washed seedlings were transferred onto paper bridges in test-tubes (25 × 150 mm) containing liquid medium with the half strength of MS salts, B5 vitamins (Gamborg et al., 1968), 100 mg l–1 myoinositol and 20 g l–1 sucrose (pH 5.6). The seedlings were grown at 28 ±2°C under 16/8 h light/dark photoperiod using white fluorescent tube light (60 µmol/m^2^/s). The positive plants were confirmed by PCR (Figure S5a-h) using the forward primer 5'-ATGATTGAACAAGATGGATTGCACG-3' (Npt-II forward primer) and the reverse primer 5'-TCAGAAGAACTCGTCAAGAAGGC-3' (Npt-II reverse primer). The seeds from the positive T1-transgenic lines were subjected to antibiotic selection (kanamycin) and successful plantlets were then transferred in the soil for hardening. In this study, the seeds collected from an individual plant were considered as one independent event. In other words, 7–10 lines selected for analysis were from 7–10 independently transformed plants of a single construct. The PCR positive and antibiotic resistant plants from the seeds of one transformed plant were considered as biological replicates.

**Yeast one-hybrid assay (Gold Y1H)**

*P_GhGDSL_* was cloned into pAbAi yeast vector using Matchmaker Gold Yeast One Hybrid (Y1H) library screening system (Clontech, USA) on *SacI* and *XhoI* restriction sites resulting in a *P_GhGDSL_*-AbAi (bait/reporter) construct, following the manufacturer’s protocol. *P_GhGDSL_*-AbAi yeast strain was obtained by integration of *P_GhGDSL_*-AbAi into the Y1H Gold yeast genome using yeast transformation protocol (Clontech, USA). The transformed colonies were spread on SD/-Ura media and incubated at 30 °C for three days. The plasmid integration was confirmed by colony PCR using Matchmaker Insert Check PCR Mix1 (Cat. No.630496, Clontech, Terra Bella, CA, USA). Thus Y1HGold (*P_GhGDSL_*/AbAi) strain and Y1HGold (p53/AbAi) control strains were obtained. For the optimization of the minimal inhibitory concentration of the antibiotic aureobasidin-A (AbA), a large healthy colony was picked from the bait and control strains and grown following the instructions (Clontech, Terra Bella, CA, USA). Of the culture, 100 μL was spread on a solid medium SD/-Ura with AbA (50 ng/mL, 100 ng/mL, 150 ng/mL, and 200 ng/mL) and SD/-Ura for control. The colonies were allowed to grow for 2–3 days at 30 °C. The minimal inhibitory concentration of AbA was determined as 100 ng/mL on SD/-Ura/AbA agar plates and SD/-Leu/AbA agar plates.

RNA was isolated from the different developmental stages (0 to 25DPA) of cotton using Spectrum™ plant total RNA kit (Cat no. STRN250, Sigma-Aldrich, St. Louis, MO, USA). The cDNA library was prepared using SMARTer® PCR cDNA synthesis kit (Cat No. 634926, Clontech, Terra Bella, CA, USA) and Advantage® 2 PCR kit (Cat No. 639207 Clontech, Terra Bella, CA, USA) as per the manufacturer’s instruction. The SMART cDNA synthesis generated cDNA ends that were homologous to the cloning site in pGADT7-Rec (prey vector). In order to identify the putative TFs, which could be interacting with the potential motifs present in the *P_GhGDSL_*, the screening using the cDNA library prepared from cotton fiber development stages along with linearized pGADT7-Rec vector was carried out. The transformed culture was spread on SD/-Leu/AbA^r^ agar plates and incubated at 37 °C. Finally, the plasmids were isolated from all the independent clones and sequencing was done with T7 sequencing primers by using the 96-capillary automated sequencer (ABI 3730xl DNA Analyzer, Carlsbad, CA, USA). Further, the constructs obtained by site-directed mutagenesis (sdm1-sdm8) and the deletion constructs (D1-D5) were prepared into yeast vector to find the MYB1 binding motif in *P_GhGDSL_* and the yeast co-transformation was performed.

**Gene co-expression network analysis of MYB1AT**

The RNA-sequencing data of *Gossypium hirsutum* from the different stages of fiber development, such as 0, 5, 10, 20, and 25 DPA, were downloaded from the NCBI sequence read archive (accession no. SRP044705). The reads were aligned to the *Gossypium hirsutum* genome by TopHat software (Trapnell et al., 2009). Cufflinks program (version 2.0.2) was used to assemble the aligned reads and to calculate the expression values (Trapnell et al., 2012). For gene co-expression network analysis, the expression values (FPKM) of 76,943 genes were used as an input file in the Cytoscape version 2.8.1 (Shannon et al., 2003). The "Expression Correlation Networks" plugin was used for the construction of a co-regulatory gene network from the genes of the different fiber developmental stages (0, 5, 10, 20, and 25 DPA). This plugin calculates positive (default r ≥ 0.95) as well as negative Pearson’s correlation coefficients (default r ≤ –0.95) among the interacting members of a network. Furthermore, the network visualization was carried out in the Cytoscape by applying the force-directed layout, where nodes (circles) in a network represent genes and the edges (links) represent the significant interaction between the expression levels of the genes across all fiber developmental stages (gene correlation network). The ggplot2 (https://cran.r-project.org/web/packages/ggplot2/) package in R version 3.1.3 was used to construct the box plot of positively and negatively interacting partners of MYB1 in different fiber developmental stages. PLACE (plant *cis*-acting regulatory DNA elements) (http://www.dna.affrc.go.jp/PLACE/) database was used for the identification of MYB1AT recognition motif (WAACCA) in the positively and negatively interacting partners and the randomly selected transcripts. In Figure S3, the heat maps actually represent the significant pathways/processes of the positively and negatively interacting genes with MYB1 at the different fiber developmental stages. Herein, we mapped the expression values (log_2_ FPKM - Fragments Per Kilobase of transcript per Million mapped reads) of co-expressing genes with MYB1. In the heat map, multiple genes were grouped into a BIN. The blue and red colors signify the positively and negatively co-expressed genes, respectively. The average statistical test followed by the Benjamini-Hochberg (FDR corrected) was used to identify functional pathways enriched in these genes.

**References**

Gamborg, O.L., Miller, R.A. and Ojima, K. (1968) Nutrient requirements of suspension cultures of soybean root cells. *Exp Cell Res* **50**:151-158.

Hofgen, R. and Willmitzer, L. (1988) Storage of competent cells for Agrobacterium transformation. *Nucleic Acids Res* **16**:9877.

Jefferson RA, Kavanagh TA and Bevan MW (1987) GUS fusions: beta-glucuronidase as a sensitive and versatile gene fusion marker in higher plants. *EMBO J* **6**:3901-3907.

Livak, K.J. and Schmittgen, T.D. (2001) Analysis of relative gene expression data using real-time quantitative PCR and the 2(-Delta Delta C(T)) Method. *Methods* **25**:402-408.

Nigam, D., Kavita, P., Tripathi, R.K., Ranjan, A., Goel, R., Asif, M., Shukla, A., Singh G, Rana, D. and Sawant, S.V. (2013) Transcriptome dynamics during fibre development in contrasting genotypes of *Gossypium hirsutum L*. *Plant Biotechnol J* **12**:204-218.

Kumar M, Singh H, Shukla AK, Verma PC and Singh PK (2013) A genotype-independent agrobacterium mediated transformation of germinated embryo of cotton (Gossypium hirsutum L.). Int J Biotechnol Res **3(1)**:91-90.

Trapnell, C., Pachter, L. and Salzberg, S.L. (2009) TopHat: discovering splice junctions with RNA-Seq. *Bioinformatics* **25**:1105-1111.

Trapnell, C., Roberts, A., Goff, L., Pertea, G., Kim, D., Kelley, D.R., Pimentel, H., Salzberg, S.L., Rinn, J.L. and Pachter, L. (2012) Differential gene and transcript expression analysis of RNA-seq experiments with TopHat and Cufflinks. *Nat Protoc* **7**:562-578.

Shannon, P., Markiel, A., Ozier, O., Baliga, N.S., Wang, J.T., Ramage, D., Amin, N., Schwikowski, B. and Ideker, T. (2003) Cytoscape: a software environment for integrated models of biomolecular interaction networks. *Genome Res* **13**:2498-2504.
